# A molecular view of the normal human thyroid structure and function reconstructed from its reference transcriptome map

**DOI:** 10.1186/s12864-017-4049-z

**Published:** 2017-09-18

**Authors:** Lorenza Vitale, Allison Piovesan, Francesca Antonaros, Pierluigi Strippoli, Maria Chiara Pelleri, Maria Caracausi

**Affiliations:** Department of Experimental, Diagnostic and Specialty Medicine, (DIMES), Unit of Histology, Embryology and Applied Biology, University of Bologna, Via Belmeloro 8, 40126 Bologna, BO Italy

**Keywords:** Human thyroid, Gene expression, Integrated transcriptome map, Meta-analysis, Human chromosome 21

## Abstract

**Background:**

The thyroid is the earliest endocrine structure to appear during human development, and thyroid hormones are necessary for proper organism development, in particular for the nervous system and heart, normal growth and skeletal maturation. To date a quantitative, validated transcriptional atlas of the whole normal human thyroid does not exist and the availability of a detailed expression map might be an excellent occasion to investigate the many features of the thyroid transcriptome.

**Results:**

We present a view at the molecular level of the normal human thyroid histology and physiology obtained by a systematic meta-analysis of all the available gene expression profiles for the whole organ. A quantitative transcriptome reference map was generated by using the TRAM (Transcriptome Mapper) software able to combine, normalize and integrate a total of 35 suitable datasets from different sources thus providing a typical reference expression value for each of the 27,275 known, mapped transcripts obtained. The experimental in vitro validation of data was performed by “Real-Time” reverse transcription polymerase chain reaction showing an excellent correlation coefficient (*r* = 0.93) with data obtained in silico.

**Conclusions:**

Our study provides a quantitative global reference portrait of gene expression in the normal human thyroid and highlights differential expression between normal human thyroid and a pool of non-thyroid tissues useful for modeling correlations between thyroidal gene expression and specific thyroid functions and diseases. The experimental in vitro validation supports the possible usefulness of the human thyroid transcriptome map as a reference for molecular studies of the physiology and pathology of this organ.

**Electronic supplementary material:**

The online version of this article (doi:10.1186/s12864-017-4049-z) contains supplementary material, which is available to authorized users.

## Background

The thyroid is the earliest endocrine structure to appear during human development. Thyroid hormones are the first detected in the human fetal circulation at 11-13 gestation weeks [1] and are necessary for the proper organism development, in particular for the nervous system and heart, normal growth and skeletal maturation. Moreover, their importance in maintaining human health through their action on metabolism and cell growth is well known [2].

The structural and functional units of the thyroid are the follicles, cystic-like structures delimited by a single-layered epithelium of follicular cells or thyrocytes of endodermal origin. The follicles contain the colloid, consisting mainly of thyroglobulin (TG), the stored form of inactive thyroid hormones [3]. The thyroid is the only endocrine gland that produces and stores its initial product and then resumes and turns it into active hormones T_4_ and T_3_, tetraiodothyronine or thyroxine and triiodothyronine, respectively. The colloid contains an amount of stored iodothyroglobulin sufficient to regulate the metabolic activity of the body for up to three months [3]. Follicular cell activity level is regulated by the thyroid-stimulating hormone or thyrotropin (TSH) produced by the anterior pituitary gland and released in turn under the control of thyrotropin-releasing hormone produced in the hypothalamus and responding, with negative feedback, to serum levels of circulating thyroid hormones T_3_ and T_4_. In the stroma among the follicles are the parafollicular cells or C-cells that produce the calcitonin hormone which lower the blood calcium by inhibiting bone resorption and renal calcium recovery [3]. The number of C-cells is far lower than that of follicular cells and was estimated to be 8.70 × 10^5^ in a standard human adult thyroid, while follicular cells were estimated to be 1.00 × 10^10^ [4, 5].

Several causes may lead to a decrease or an increase of thyroid function with the clinical pictures of hypo- or hyperthyroidism [6]. In particular, congenital hypothyroidism, the most frequent endocrine congenital disease, can occur either based on a thyroid hormone biosynthesis defect or predominantly due to thyroid dysgenesis [6]. In persons affected by Down syndrome (DS), in which the trisomy of chromosome 21 leads to an altered gene expression condition, the thyroid function is impaired in at least 15% of cases and usually it is a congenital hypothyroidism that is approximately 28 times more common in DS than in disomic subjects [7].

The thyroid gland is also the endocrine tissue most affected by cancer and many studies have been performed to link gene signatures with thyroid dysfunction or tumors [8–10]. Many studies typically concern the transcriptome of thyroid cancer [11], and in these cases the normal thyroid tissue often used as a control was derived from apparently healthy tissue of diseased gland (histologically normal thyroid tissue adjacent to thyroid tumor).

Transcriptome studies are able to provide a representation of gene expression at the molecular level, thus underlying pathways or genes with specific functions in a given tissue [12]. Alteration of the reference gene expression level could be then related to a particular pathologic process, with implications for diagnosis, prognosis and identification of molecular targets for therapy [13].

Human studies of gene expression profile in normal thyroid are limited and have been conducted on a variety of experimental platforms by different Authors over many years. Gene expression profiles of whole normal thyroid can be found in the public databases in the context of normal human gene expression studies.

**Fig. 1:**
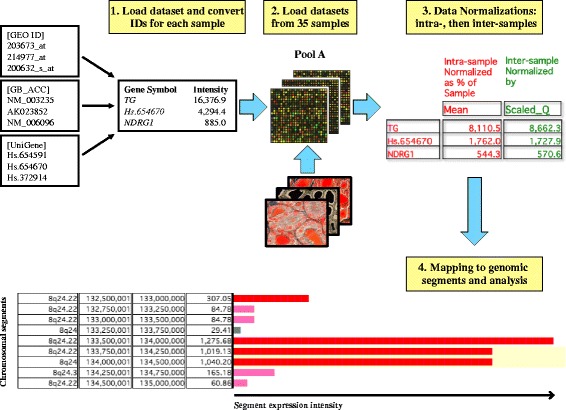
Graphic representation of the TRAM software workflow (simplified) for the study of thyroid transcriptome (Pool A). Gene expression datasets obtained by any sample of interest in tab-delimited text format are imported, probe names are assigned to individual loci following conversion of all types of gene identifiers (IDs) into official gene symbols, raw gene expression values are intra-sample normalized as percentage of the sample mean value and inter-sample normalized by scaled quantile. The final reference value for each locus is the mean value of all available normalized values for that locus. The expression is finally also mapped along each chromosome and graphically displayed as expression intensity for each chromosomal segment, expressed as the mean of the expression values of the loci included in that segment. Over- and underexpressed regions are then determined following statistical analysis. If Pools A and B are compared, the values would represent the A/B ratios

The aim of this study was to perform a systematic meta-analysis of the gene expression profiling experiments on the whole normal human thyroid provided by the European Bioinformatics Institute (EBI) [14] and the National Center for Biotechnology Information (NCBI) [15] databases (named ArrayExpress and GEO (Gene Expression Omnibus), respectively) in order to generate a quantitative transcriptome reference map of the whole gland. RNA microarrays and RNA sequencing (RNA-Seq) are considered as the two main kinds of high-throughput technologies to study the gene expression profile [13], and it was demonstrated that comparison between microarray based quantification and RNA-Seq quantification showed concordance between the two technologies [16]. Due to the very small number of RNA-Seq studies available for the whole adult normal human thyroid (many experiments are performed on non-coding RNA expression, on fetal thyroid transcriptome or on cell lines), we have selected datasets obtained by expression microarrays published online from 2002 to 2013 for this study. Thirty-five suitable gene expression profile datasets found from different sources were combined and integrated using TRAM (Transcriptome Mapper) software [17], which is able to generate transcriptome maps from any source listing gene expression values for a given gene set, e.g. expression microarray, thus allowing a typical reference value of expression for each of the known and uncharacterized (unmapped) transcripts assayed by any of the experimental platforms used in this regard to be obtained (Fig. 1). In addition, our goal was to realize a graphical representation of gene expression profiles along the chromosomes to analyze transcription intensity in specific chromosomal regions (Fig. 1).

Experimental validations has been performed to test if our map may provide a general reference quantitative model of the thyroid transcriptome. Finally, a quantitative gene expression map offers the possibility to highlight differential expression between normal human thyroid and a pool of non-thyroid tissues and we also tested if this may provide data useful to model correlations between thyroidal gene expression and specific thyroid functions and diseases.

## Results

### Database search and database building

We retrieved 35 samples from 10 microarray experiments performed on the whole normal thyroid excluding histologically normal samples recovered from tissue adjacent to thyroid cancer. Sample identifiers and main sample features are listed in the Additional file 1 (thyroid, male thyroid, female thyroid) and Additional file 2 (pool of non-thyroid tissues) (both datasets are available at: http://apollo11.isto.unibo.it/suppl).

The pool of non-thyroid tissue samples (624 samples from 12 microarray experiments) was already used in [12], except for the inclusion of brain samples and exclusion of thyroid samples in the comparison Pool B. Datasets were loaded into TRAM and analyzed obtaining five transcriptome maps: thyroid (Pool A); thyroid (Pool A) vs. pool of non-thyroid tissues (Pool B); male thyroid (Pool A.1); female thyroid (Pool A.2); male thyroid (Pool A.1) vs. female thyroid (Pool A.2).

### Thyroid and thyroid vs. pool of non-thyroid tissues transcriptome map analysis

Each map provides data as previously described [18]. Briefly, a TRAM map provides a reference gene expression value for all human mapped loci following intra-sample normalization (the raw value is expressed as percentage of the mean value for that sample) and inter-sample normalization (the value is further normalized by the quantile method to provide the mean value among the expression values available for all samples and having the same rank when each profile is ordered by descending order of these values) [17]. To maximize the data that may be extracted from diverse experimental platforms in a cross-platform model, overcoming the typical limitation of the standard quantile method requiring each platform provides the same number of genes/values, we applied the scaled quantile method. This allowed the normalization of data derived from platforms with a highly different number of probes by adjusting the rank for each value in a sample in proportion to the sample having the maximum number of values, so effectively averaging highest/lowest values of a sample with the highest/lowest values of the other samples [17]. The final result is a reference expression value for a locus summarizing each available data point, allowing the comparison between two biological conditions when reference values for a given locus are present in both (A and B) sample pools considered, in the form of A/B ratio. In addition, the physical location based analysis highlights when the segment expression value (mean expression values of the genes contained in a 500 kb genomic segment) is found to be statistically significantly over−/underexpressed by hypergeometric distribution method in the comparison between the two sample sources [17]. Gene content of each over−/underexpressed genomic segment was further checked to exclude segments containing over−/underexpressed genes whose expression value resulted from less than five data points in at least one of the two compared pools [18]. A segment or a gene was considered to be statistically significantly over−/underexpressed for q < 0.05, where q is the *p*-value obtained by the method of hypergeometric distribution [17] and corrected for multiple comparisons. When the results were reported for the over−/underexpressed single genes, the segment was set to 12.5 kb and we considered only the genes associated with at least five data points [18].

Detailed results for each map are provided below and are also available at: http://apollo11.isto.unibo.it/suppl.

In the thyroid transcriptome map analysis, 933,461 data points corresponding to 27,275 mapped loci were included (Additional file 3). 11,620 data points of the map corresponded to 352 chromosome 21 (chr21) mapped loci. Results obtained by analysis included 31 significantly overexpressed segments, some of which are partially overlapped (see TRAM results file available at http://apollo11.isto.unibo.it/suppl). The genome segments having the highest statistically significant expression values are shown in Table 1. The first one is on chromosome 8 (8q24) and includes the known genes *TG* and *NDRG1* (N-myc downstream regulated 1). The second overexpressed segment includes many members of the metallothionein gene family. There are no statistically significant underexpressed genomic segments.

**Table 1:** The first five genomic segments significantly over−/underexpressed in the Thyroid (Pool A) transcriptome map and in the Thyroid (Pool A) vs. Pool of non-thyroid tissues (Pool B) transcriptome map

**Thyroid transcriptome map**					
Chr^a^ and Location	Segment Start^b^	Segment End^b^	Value A	q-value	Genes in the segment^c^
Overexpressed segments					
chr8 8q24.22	134,000,001	134,500,000	1,040.20	0.0033	*TG*, Hs.654670, *NDRG1*
chr16 16q12.2	56,500,001	57,000,000	907.10	< 0.0001	*MT2A*, *MT1E*, *MT1A*, *MT1F*, *MT1G*, *MT1H*, *MT1X*, *HERPUD1*
chr1 1p21	103,750,001	104,250,000	768.87	0.0009	*AMY2B*, *AMY2A*, *AMY1B*
chr11 11q13.1	65,000,001	65,500,000	763.28	0.0001	*NEAT1*, Hs.736281, Hs.593027, *MALAT1*, Hs.712678, Hs.732685
chr4 4q13	70,500,001	71,000,000	689.99	0.0021	*STATH, HTN3, HTN1*
**Thyroid vs. Pool of non-thyroid tissues transcriptome map**					
Chr^a^ and Location	Segment Start^b^	Segment End^b^	Value A/B	q-value	Genes in the segment^c^
Overexpressed segments					
chr8 8q24	134,000,001	134,500,000	30.57	0.0024	*TG*, Hs.654670, *ST3GAL1*
chr14 14q13.2-q13.3^d^	36,500,001	37,000,000	13.47	< 0.0001	*MBIP*, *SFTA3*, Hs.715763, *NKX2-1*
chr6 6q25.1^d^	150,250,001	150,750,000	11.15	0.0005	Hs.15422, *PPP1R14C*, *IYD*
chr2 2q12-q14.1	113,750,001	114,250,000	6.76	0.0045	*PAX8*, Hs.656634, Hs.675465
chr8 8q23	110,500,001	111,000,000	5.17	< 0.0001	*PKHD1L1*, Hs.88651, *EBAG9*, Hs.632960
Underexpressed segments					
chr2 2p12	79,000,001	79,500,000	0.17	0.0001	*REG1B*, *REG1A*, *REG3A*
chr18 18q12.1	28,500,001	29,000,000	0.27	0.0002	*DSC3*, Hs.557559, *DSC1*, *DSG1*
chr1 1q21-q22	153,000,001	153,500,000	0.29	< 0.0001	*SPRR1B*, *SPRR2D*, *SPRR2A*, *LELP1*, *LOR*, *S100A*, *S100A7*
chr5 5q32	147,250,001	147,750,000	0.30	< 0.0001	*SCGB3A2*, *C5orf46*, *SPINK5*, *SPINK7*
chr10 10q26.11	118,000,001	118,500,000	0.33	0.00087	*PNLIP*, *PNLIPRP1*, *PNLIPRP2*

Analysis was performed using default parameters (see Methods section). Overexpressed segments are sorted by decreasing expression values in the thyroid transcriptome map; underexpressed segments are sorted by increasing expression values. In the ‘Map’ mode, TRAM displays UniGene EST clusters (with the prefix “Hs.” in the case of *H. sapiens*) only if they have an expression value. For simplicity, some segments are not shown because they overlap with those highlighted in one of the listed regions. In bold the genes with a statistically significant over- or underexpression values in the segment. The complete results for these models are available as online supplementary material (see text). ^a^Chromosome. The segment location cytoband was derived from that of the first mapped gene within the segment. ^b^Segment Start/End: chromosomal coordinates for each segment. ^c^Significantly over-/underexpressed genes as marked in the TRAM results. ^d^Cytoband not available in Gene was derived from the UCSC Genome Browser (http://genome-euro.ucsc.edu/cgi-bin/hgGateway)

At the single gene level the known gene presenting the highest expression value (8,662.34) is still *TG*, but the EST (expressed sequence tag) cluster Hs.732685 appears to have the highest absolute expression value (10,763.93). *TG* is followed by *RPL41* (5,903.29) and *TPT1* (5,743.83), encoding for the ribosomal protein L41 and for the tumor protein, translationally-controlled 1, respectively (Table 2). The lowest expression value belongs to the EST cluster Hs.734567 (2.95). The other genes with the highest and lowest expression values are shown in Table 2 and Additional file 3. Among the known genes of chromosome 21, *TFF3*, encoding for trefoil factor 3, has the highest expression value (2,726.93) (Table 2).

**Table 2:** List of the five most over- and underexpressed genes (all significantly, with q < 0.05) in the Thyroid (Pool A) transcriptome map

Gene name	Value A	Location	Data points A	SD as % of expression A
Overexpressed genes				
Hs.732685^a^	10,763.93	11q13.1	11	23.12
* TG*	8,662.34	8q24	33	33.14
* RPL41*	5,903.29	12q13	32	51.15
* TPT1*	5,743.83	13q14	101	42.11
* EEF1A1*	5,537.45	6q14.1	106	59.81
Overexpressed chr21 genes				
* TFF3*	2,726.93	21q22.3	36	78.82
Hs.709790	1,358.90	21q22.11	18	56.76
* CSTB*	1,271.11	21q22.3	33	123.06
* SOD1*	1,156.80	21q22.11	37	59.00
* PCP4*	848.72	21q22.2	33	70.73
Underexpressed genes				
Hs.734567^b^	2.95	12p13.2	11	50.79
* HTR3E*	3.08	3q27.1	8	28.12
Hs.591590^b^	3.32	2p12	11	60.32
LOC645739	3.40	13q12.11	9	27.64
* ZNF852*	3.45	3p21.31	11	56.87
Underexpressed chr21 genes				
Hs.434909^b^	4.32	21q22.12	11	48.95
Hs.555591^b^	4.65	21q21.2	11	49.55
Hs.27261^b^	4.86	21q22.13	11	89.74
* KRTAP19-4*	5.21	21q22.1	11	48.26
LOC339622	5.29	21q21.1-q21.2	11	102.47

‘Value’: mean gene expression value normalized across all the pool samples; ‘Data points’: number of spots associated to an expression value for the locus; ‘SD’: standard deviation for the expression value expressed as a percentage of the mean. Overexpressed genes are sorted by decreasing gene expression value, underexpressed genes are sorted by increasing gene expression value. Full results are available as supplementary material (see text). ^a^According to the criteria detailed in the Methods section, the segment window (12,500 bp) contains more than one over−/underexpressed gene, but the significance is assumed to remain because the expression value of this over−/underexpressed gene prevails over the others. ^b^Cytoband not available in Gene was derived from the UCSC Genome Browser (http://genome-euro.ucsc.edu/cgi-bin/hgGateway)

In the analysis of the thyroid vs. pool of non-thyroid tissues transcriptome map, regional differential expression of Pool A (35 thyroid samples) versus Pool B (624 non-thyroid tissues samples) for a total of 26,750 analyzed loci with values for both Pools A and B, was investigated. Results obtained by analysis include 34 significantly over−/underexpressed segments: 22 of them are overexpressed and 12 are underexpressed. The genome segment that has the highest statistically significant expression ratio is on chromosome 8 (8q24) (Table 1) including again the known gene *TG* and also *ST3GAL1* gene encoding for ST3 beta-galactoside alpha-2,3-sialyltransferase 1 and the EST cluster Hs.654670. The following overexpressed segments contain genes having a key role in thyroid functioning as *NKX2-1* (NK2 homeobox 1), *IYD* (iodotyrosine deiodinase) and *PAX8* (paired box 8) (Table 1). The genome segment that has the lowest statistically significant expression ratio is on chromosome 2 (2p12), including the known genes *REG1B*, *REG1A* and *REG3A*, encoding for regenerating family member 1 beta, 1 alpha and 3 alpha, respectively (Table 1).

At the single gene level, an increase of more than 10 times of the gene expression ratio was observed in all the first 51 loci when ordered by decreasing values of thyroid/non-thyroid expression ratio, mostly including known genes with thyroid function. Notably, the first two overexpressed genes in thyroid in comparison with non-thyroid tissues are *TG* (ratio = 188.22) and *TPO* (thyroid peroxidase) (ratio = 151.17) followed by *SLC26A7* (solute carrier family 26 member 7) (ratio = 66.94), *IYD* (ratio = 66.25) and *TSHR* (thyroid stimulating hormone receptor) (ratio = 49.36) (Table 3 and Additional file 4). We also observed that in this range of expression ratios, one chr21 known gene is included: *TFF3* (ratio = 16.14). Among the genes with the lowest A/B expression ratio, a 100-fold decrease (ratio = 0.01) was observed for the gene *LCE5A*, encoding for the late cornified envelope 5A (Table 3).

**Table 3:** List of the five (or more) most over- or underexpressed genes (all significantly, with q < 0.05, except genes with ^a^ note) in the Thyroid (Pool A) vs. Pool of non-thyroid tissues samples (Pool B) transcriptome map

Gene name	Value A	Value B	Ratio A/B	Location	Data points A	Data points B	SD as % of expression A	SD as % of expression B
Overexpressed genes								
* TG*	8,662.34	46.02	188.22	8q24	33	566	33.14	221.41
* TPO*	2,245.11	14.85	151.17	2p25	35	560	54.57	118.35
Hs.654670^ab^	1,727.93	12.30	140.54	8q24	18	413	86.71	108.60
* SLC26A7*	1,107.90	16.55	66.94	8q23	53	1,198	132.82	226.70
* IYD*	890.07	13.44	66.25	6q25.1	24	456	65.51	137.47
Hs.656634^ab^	1,982.24	39.54	50.14	2q13	18	413	80.05	101.09
* TSHR*	487.64	9.88	49.36	14q31	92	1,907	91.14	130.07
Overexpressed chr21 genes								
* TFF3*	2,726.93	168.96	16.14	21q22.3	36	758	78.82	210.27
Hs.129583^b^	273.20	24.62	11.10	21q22.13	11	296	51.98	69.50
* DNMT3L*	101.54	13.76	7.38	21q22.3	31	449	357.89	107.64
* IFNAR1*	272.52	45.93	5.93	21q22.11	65	1,405	491.61	75.21
Hs.709790^ab^	1,358.90	231.48	5.87	21q22.11	18	418	56.76	78.65
Under-expressed genes								
* LCE5A*	24.09	1,639.23	0.01	1q21.3	11	30	30.42	39.05
* MSMB*	9.44	527.57	0.02	10q11.2	53	1,152	124.26	347.24
* PRM2* ^a^	6.51	309.26	0.02	16p13.2	44	553	49.00	530.15
* KRTDAP*	11.24	480.77	0.02	19q13.12	16	454	39.24	354.39
* ORM2*	6.19	255.66	0.02	9q32	21	529	38.82	477.48
Underexpressed chr21 genes								
* S100B*	15.70	259.61	0.06	21q22.3	44	865	111.12	324.61
* TFF1*	22.66	184.51	0.12	21q22.3	33	652	108.08	280.42
* KRTAP10-10*	14.16	91.19	0.16	21q22.3	16	30	46.04	92.21
* OLIG1*	12.59	76.13	0.17	21q22.11	24	481	59.64	350.79
* TPTE*	11.07	53.19	0.21	21p11	21	559	76.74	465.37

‘Value’: mean gene expression value normalized across all the pool samples; ‘Data points’: number of spots associated to an expression value for the locus; ‘SD’: standard deviation for the expression value expressed as a percentage of the mean. Overexpressed genes are sorted by decreasing gene expression ratio, underexpressed genes are sorted by increasing gene expression ratio. Full results are available as supplementary material (see text). ^a^This gene is one of the five most over−/underexpressed ones, but the value is not statistically significant. This is either because this gene was present with other genes even in the 12,500 bp segment and its expression value does not prevail over the others, or because the segment containing the gene does not fulfil criteria for local over−/underexpression ^b^Cytoband not available in Gene was derived from the UCSC Genome Browser (http://genome-euro.ucsc.edu/cgi-bin/hgGateway)

When there are more than five genes listed, it is because we believe it is important to highlight them

The availability of complete TRAM datasets allows to manually check for single loci of interest if the high standard deviation (e.g., SD > 100% of the mean expression value) calculated by TRAM for each locus might arise from: physiological inter-individual (inter-sample) variation; imprecision in the measurement while approaching the lowest range of detection (i.e. expression values < 50); artifacts due to the fact that in some experimental platforms different probes located on the array have been assigned to the same locus but they actually detect different transcripts with systematically consistent highly different measures for each of the probes.

### Male thyroid vs. female thyroid transcriptome map analysis

The thyroid samples found as described above and for which the sex of the sample donor was available were grouped into two additional datasets: Pool A.1 including 6 male samples and Pool A.2 including 12 female samples (Additional file 1), and the corresponding transcriptome maps were generated and compared.

The gene expression value for each of the 25,954 loci of the male vs. female thyroid differential transcriptome map is available providing for each of them the respective gene expression ratio (Additional file 5, supplementary full results for transcriptome maps are available at: http://apollo11.isto.unibo.it/suppl/).

At the single gene level, a more than 10-fold increase of the gene expression ratio was observed only for the first locus *KDM5D* on chromosome Y encoding for the lysine demethylase 5D. In particular, a more than 8-fold increase was observed for the known genes *DDX3Y* (8.74), *USP9Y* (8.50), both on Y, and *TAS2R38* (8.25), encoding respectively for DEAD-box helicase 3, ubiquitin specific peptidase 9, Y-linked, and for taste 2 receptor member 38 on chr7 (Additional file 5). Among the genes with the lowest A.1/A.2 expression ratio, the *XIST* gene (X inactive specific transcript, on chrX) was the fifth with a 26.71-fold decrease (ratio = 0.04) (Additional file 5).

**Fig. 2:**
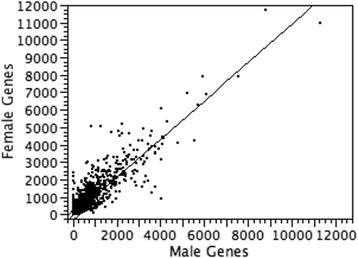
Bivariate analysis between the gene expression values of the thyroid transcriptome map in male and in female cells. The fit line is shown; Pearson correlation coefficient is 0.92 and *p*-value < 0.0001

Bivariate analysis of the statistical correlation between the gene expression values of the thyroid transcriptome map in male and in female cells was performed (Fig. 2). The results showed a significant statistical correlation of data (*r* = 0.92, *p*-value < 0.0001), confirming the large overlapping of results between the two transcriptome maps, with the exception of single genes with a well-known sex-biased expression pattern.

When sorting by Pool A.1 (male thyroid) expression values, the most expressed genes have a similar expression pattern in both sexes with differential ratios close to 1 (Additional file 6).

### Housekeeping gene search

In the thyroid transcriptome map, the search for housekeeping genes with the described criteria (Methods section) retrieved 45 loci, including *BLOC1S2* (biogenesis of lysosomal organelles complex 1 subunit 2) which is the known gene that has the lowest standard deviation (SD = 13.39 expressed as percentage of the mean value), and the known genes *ACTG1* and *RPLP0*, encoding for actin gamma 1 and ribosomal protein lateral stalk subunit P0, respectively, having the highest number of data points (*n* = 180 and *n* = 76) (Table 4) and an excellent ratio between the Pools A and B (1.04 and 0.99).

**Table 4:** Predicted housekeeping genes from the thyroid transcriptome map (explanation in the text); (*n* = 45)

Gene symbol	Chromosome	Location	Expression value	Data points	SD
Hs.670607	chr4	N/A	232.70	18	11.95
*BLOC1S2*	chr10	10q24.31	181.44	22	13.39
Hs.706927	chr6	N/A	357.37	18	13.82
*TMEM256*	chr17	17p13.1	153.91	22	18.35
*POM121C*	chr7	7q11.2	347.50	20	22.49
*CUEDC2*	chr10	10q24.32	210.93	31	22.83
RPLP0*	chr12	12q24.2	**3,340.82**	**76**	**23.40**
*PJA1*	chrX	Xq13.1	234.54	31	23.83
Hs.713747	chr1	N/A	169.93	18	23.92
*RAB25*	chr1	1q22	199.48	31	24.00
*CIB1*	chr15	15q25.3-q26	314.61	33	25.01
*MRPL18*	chr6	6q25.3	182.80	23	25.09
*SCAMP3*	chr1	1q21	152.98	33	25.11
Hs.633993	chr8	N/A	2,050.15	18	25.21
Hs.714416	chr3	N/A	100.67	18	25.31
*RBM23*	chr14	14q11.2	257.95	31	25.67
Hs.117688	chr8	N/A	160.32	22	25.73
Hs.546523	chr2	N/A	178.40	18	25.96
*DCXR*	chr17	17q25.3	210.53	31	25.98
Hs.193784	chr3	N/A	491.90	22	26.02
*ABRACL*	chr6	6q24.1	116.71	23	26.15
*MAP1LC3B2*	chr12	12q24.22	157.45	18	26.29
*DGCR6*	chr22	22q11	209.18	20	26.31
*GTPBP6*	chrX; chrY	Xp22.33; Yp11.32	120.83	23	26.61
*AIMP2*	chr7	7p22	181.19	43	26.84
*CSNK2B*	chr6	6p21.3	337.74	25	26.89
*TM2D2*	chr8	8p11.22	256.54	28	27.09
*CLIC1*	chr6	6p21.3	530.89	25	27.21
Hs.715132	chrX	N/A	196.12	22	27.42
Hs.732277	chr19	N/A	218.68	22	28.49
Hs.705664	chr7	N/A	1,167.55	18	28.62
*NMRAL1*	chr16	16p13.3	110.71	45	28.70
Hs.713819	chr5	N/A	241.81	18	28.93
*RPP21*	chr6	6p22.1	111.91	23	28.94
*PNMA1*	chr14	14q24.3	148.75	23	28.94
ACTG1*	chr17	17q25	**3,583.46**	**180**	**29.06**
*AKR1B1*	chr7	7q35	258.94	25	29.09
*APOA1BP*	chr1	1q21	231.69	32	29.35
*MRPL24*	chr1	1q23.1	183.95	31	29.44
*ABCF1*	chr6	6p21.33	123.95	25	29.56
*ZNF627*	chr19	19p13.2	112.37	24	29.70
*METRNL*	chr17	17q25.3	175.03	22	29.78
*OCIAD2*	chr4	4p11	286.18	20	29.92
*EIF2B2*	chr14	14q24.3	248.05	33	29.94
*DIABLO*	chr12	12q24.31	211.60	31	29.98

Genes are sorted in ascending order of SD as percentage of the mean value. *In bold, the two best genes at behaving like a housekeeping gene due to a combination of a low SD, a high expression value and a high number of data points, following checking in the “Values A” TRAM table that the 180 (*ACTG1*) and 76 (*RPLP0*) data points are derived from 33 and 22 samples, respectively, out of the 35 thyroid samples analyzed. N/A: not available in the Gene database (http://www.ncbi.nlm.nih.gov/gene) when the analysis was performed

### Gene expression level in the thyroid and association to thyroid mutant phenotypes

We chose to analyze the first 50 known genes of the thyroid vs. non-thyroid tissue differential transcriptome map, sorted in decreasing order of the A/B ratio, in order to find an association to thyroid phenotypes registered in OMIM (Online Mendelian Inheritance in Man). We compared the set of the first 25 vs. the second set of 25 known genes through Fisher’s exact test and we found a statistically significant greater number of genes associated with thyroid phenotype in the first set of 25 known genes, 32% of the genes (8 out of 25, with A/B ratio from 13.91 (*FOXE1*, forkhead box E1) to 188.22 (*TG*)) rather than 4% of the second group (one out of 25 (*DUOX2*, dual oxidase 2, ratio A/B = 7.26)) (*p* = 0.023) (Table 5).

**Table 5:** Association of the first 50 known genes more expressed in thyroid respect to non-thyroid pool tissues (ratio between Pools A and B) to OMIM phenotypes in the case of gene mutation

# Gene		A/B Value	OMIM-Phenotype	OMIM-Phenotype ID
A)				
**1**	***TG***	**188.22**	**Thyroid Dyshormonogenesis 3 (AR)**	**#274700**
**2**	***TPO***	**151.17**	**Thyroid Dyshormonogenesis 2A (AR)**	**#274500**
3	*SLC26A7*	66.94	-	N/A
**4**	***IYD***	**66.25**	**Thyroid Dyshormonogenesis 4 (AR)**	**#274800**
**5**	***TSHR***	**49.36**	**Hyperthyroidism, familial gestational**	**#603373**
			**Hyperthyroidism, nonautoimmune (AD)**	**#609152**
			**Hypothyroidism, congenital, nongoitrous, 1 (AR)**	**#275200**
			Thyroid adenoma, hyperfunctioning, somatic	N/A
			Thyroid carcinoma with thyrotoxicosis, somatic	N/A
**6**	***SLC26A4***	**44.46**	**Pendred syndrome (AR)**	**#274600**
7	*SNORA26*	40.26	-	N/A
8	*PRH2*	37.53	-	N/A
9	*SLC26A4-AS1*	36.09	not in OMIM	
10	*SFTA3*	34.92	not in OMIM	
11	*HIST2H4B*	32.52	not in OMIM	
12	*HSP90AA2*	19.71	-	N/A
**13**	***NKX2-1***	**18.20**	**Choreoathetosis, hypothyroidism, and neonatal respiratory distress (AD)**	**#610978**
			**Thyroid cancer, monmedullary, 1 (AD)**	**#188550**
14	*LIPG*	17.93	-	N/A
15	*C16orf89*	17.27	not in OMIM	
**16**	***DIO1***	**15.19**	**Hyperthyroxinemia (AD)**	**+147,892**
**17**	***FOXE1***	**13.91**	**Athyroidal hypothyroidism, with spiky hair and cleft palate**	**#241850**
			**Bamforth-Lazarus syndrome) (AR)**	
			**Thyroid cancer, nonmedullary, 4 (AD)**	**#616534**
18	*TNFRFS11B*	13.52	-	N/A
19	*RMST*	13.14	-	N/A
20	*HHEX*	12.93	-	N/A
21	*SNORD22*	12.16	-	N/A
22	*INPP5J*	11.14	-	N/A
23	*CLIC3*	9.99	-	N/A
24	*OTOS*	9.19	-	N/A
25	*SLC25A29*	9.09	-	N/A
B)				
26	*SGK223*	8.99	not in OMIM	
27	*ID4*	8.97	-	N/A
28	*PDE8B*	8.84	-	N/A
29	*LRP2*	8.42	-	N/A
30	*ZBED2*	8.33	-	N/A
31	*ST6GAL2*	8.14	-	N/A
32	*DIO2*	8.00	-	N/A
33	*CLDN3*	7.39	-	N/A
**34**	***DUOX2***	**7.26**	**Thryoid dyshormonogenesis 6 (AR)**	**#607200**
35	*MGAT4C*	7.04	-	N/A
36	*IPCEF1*	6.93	not in OMIM	
37	*COL23A1*	6.88	-	N/A
38	*CRABP1*	6.78	-	N/A
39	*CYS1*	6.51	not in OMIM	
40	*SHISA2*	6.46	not in OMIM	
41	*PKHD1L1*	6.36	-	N/A
42	*WDR72*	6.35	-	N/A
43	*TCERG1L*	6.19	not in OMIM	
44	*MPPED2*	6.18	-	N/A
45	*IGFBPL1*	6.13	-	N/A
46	*NEUROG2*	6.13	-	N/A
47	*GABRA6*	6.08	-	N/A
48	*ID3*	5.75	-	N/A
49	*HIRA*	5.66	-	N/A
50	*MT1F*	5.49	-	N/A

Gene symbols related to thyroid phenotypes are in bold, as well as the related phenotypes. A) the first 25 genes with the highest A/B ratio (>9 folds); B) the second group of 25 more differentially expressed genes with A/B ratio ranging from 8.99 to 5.49 folds. Genes with data points < 5 and SD as % of the mean value >200 were excluded from the analysis. AR: autosomal recessive; AD: autosomal dominant

### Validation of thyroid transcriptome map results through Real-Time RT-PCR (reverse transcription-polymerase chain reaction)

In order to confirm the results of our meta-analysis study, we validated the transcriptome map of whole thyroid performing some Real-Time RT-PCR assays.

**Fig. 3:**
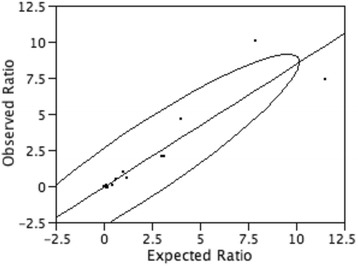
Bivariate analysis between observed (Real-Time RT-PCR) and expected (TRAM) expression ratios of 16 selected genes (listed in Table 6; *REEP3* resulted to be undetectable) in the thyroid transcriptome map. The fit line is shown; Pearson correlation coefficient is 0.93 and *p*-value < 0.0001

Using criteria as described in the Methods section, we selected 17 genes from the thyroid transcriptome map: *TG*, *RPL41, B2M, ACTB*, *TPO, IYD, SDC2*, *TSHR, SERPINF1, MYH9*, *BACE2, REEP3*, *YKT6*, *NTNG1, RIPPLY3, NPTX1, TBX18* (Table 6). The *SDC2* gene was chosen as the reference gene in that its predicted intermediate level of expression to avoid artifacts more likely to happen while approaching extremely low or high values. The primer pairs used to validate results are listed in the Additional file 7. The in vitro observed gene expression ratios between each target gene and the reference gene are provided in Table 6. The correlation between the observed and expected gene expression ratios as calculated by bivariate analysis was statistically highly significant with the Pearson correlation coefficient being 0.93 and *p*-value < 0.0001 (Fig. 3).

**Table 6:** Selected genes to validate the thyroid transcriptome map in vitro through Real-Time RT-PCR

Gene symbol	Gene name	EEV	SD	ER	Ct mean	OR
*TG*	Thyroglobulin	8,662.34	33.14	11.51	17.55	7.41
*RPL41*	Ribosomal protein L41	5,903.29	51.15	7.85	17.11	10.056
*B2M*	Beta-2-microglobulin	2,988.28	64.22	3.97	18.22	4.66
*ACTB*	Actin, beta	2,338.95	63.50	3.11	19.38	2.085
*TPO*	Thyroid peroxidase	2,245.11	54.57	2.98	19.38	2.08
*IYD*	Iodotyrosine deiodinase	890.07	65.51	1.18	21.16	0.61
***SDC2***	**Syndecan 2**	**752.49**	**59.44**	**1**	**20.44**	**1**
*TSHR*	Thyroid stimulating hormone receptor	487.64	91.14	0.65	21.34	0.54
*SERPINF1*	Serpin family F member 1	320.48	65.03	0.43	23.63	0.11
*MYH9*	Myosin, heavy chain 9, non-muscle	315.95	104.13	0.42	24.19	0.074
*BACE2*	Beta-site APP-cleaving enzyme 2	105.67	47.06	0.14	24.75	0.050
*REEP3*	Receptor accessory protein 3	85.88	108.64	0.11	N/A	N/A
*YKT6*	YKT6 v-SNARE homolog (*S. cerevisiae*)	29.63	96.23	0.04	26.08	0.020
*NTNG1*	Netrin G1	17.89	83.44	0.024	32.51	2.33 × 10^−4^
*RIPPLY3*	Ripply transcriptional repressor 3	16.88	97.44	0.022	33.10	1.55 × 10^−4^
*NPTX1*	Neuronal pentraxin 1	16.77	79.37	0.022	33.37	1.28 × 10^−4^
*TBX18*	T-box 18	11.52	100.73	0.015	28.63	0.0034

In bold the gene chosen as reference. From left to right: official gene symbol of the selected gene; official full gene name; expected expression value (EEV), i.e. mean linear expression value for the gene as provided by TRAM software; standard deviation (SD) as percentage of the mean; expected ratio (ER) between target and reference gene expression value; threshold cycle (Ct) mean provided by BioRad CFX Manager Software 2.1 manually positioning the threshold line; observed ratio (OR) determined between each target gene and the reference gene using the 2^∆Ct^ method, according to the formula: 2^ΔCt^ = 2 ^Ct ref. - Ct target^

## Discussion

### Whole normal human thyroid transcriptome map

The human thyroid is an extremely differentiated organ performing exclusive functions related in particular to the production of hormones with a variety of actions indispensable for normal development, growth and function of the organism. This particularity is well reflected in classic dissertations about the thyroid in the fields of biochemistry [19], histology [20], anatomy [3] and physiology [21], where typical proteins originating from the thyroid are considered a marker of the organ. Modern technologies both in molecular and computational biology now make possible a quantitative, global representation of the whole gene transcription profile in a cell type which is not biased by previous knowledge, but allows systematic reconstruction of the critical aspects of gene expression at a genomic level.

We present here a quantitative transcriptome reference map of the whole normal human thyroid, i.e. a reference typical value of expression for each of the 27,275 known, mapped and 13,002 uncharacterized (unmapped) transcripts. Available gene expression data from a relevant number of samples and different sources were integrated through the TRAM software [17], resulting in a gene expression atlas of the whole gland useful as a “generally applicable” transcriptome model of normal thyroid. Actually, the TRAM approach has been very recently used with success in comparing normal vs. pathological conditions in colon cancer [22] and Parkinson’s disease [23], therefore possible conceivable applications of the atlas we provided here include quantitative, systematic comparisons of this profile with the one obtained from thyroid normal-near-tumor samples as well as tumor samples in further works on the subjects.

TRAM software analysis provides a segment representation of the genome that could help to identify chromatin domains whose genes are encoding for proteins interacting with each other or belonging to the same cell pathway [17].

The transcriptome maps showed an excellent portrait of thyroid physiology, in fact the first overexpressed segment is on chr8 (Table 1) and includes the known genes *TG* and *NDRG1*. The first gene encodes for the form of thyroid hormone storage, the major product of thyroid gland, accumulated in large quantities in the lumen of thyroid follicles. The expression of the second gene is increased in thyroid carcinomas with respect to normal and benign thyroid lesions and is correlated with more advanced tumor stages [24]. *NDRG1* is a member of the N-myc downregulated gene family (alpha/beta hydrolase superfamily) and encodes for a cytoplasmic protein involved in stress responses, hormone responses, cell growth, and differentiation, necessary for p53-mediated caspase activation and apoptosis (see NCBI Gene entry https://www.ncbi.nlm.nih.gov/gene/10397). The same segment also includes the EST cluster Hs.654670 already found to be highly expressed in the thyroid, according to its UniGene EST profile, and whose transcript is increased ~ 141 fold in thyroid compared to non-thyroid tissues according to our map. This segment could represent a previously unknown cluster of genes converging on specialized thyroid functions and where physical proximity could suggest a coregulation of expression.

The second segment overexpressed is on chr16 (Table 1) and includes the gene cluster of metallothioneins: *MT2A*, *MT1E*, *MT1A*, *MT1F*, *MT1G*, *MT1H* and *MT1X*. Metallothioneins are cytoprotective proteins acting as scavengers of toxic metal ions or reactive oxygen species. They are upregulated in follicular thyroid carcinoma and are regarded as a marker of thyroid stress in Graves’ disease [25]. The Authors supposed that TSH represents a key regulator of metallothionein expression in thyrocytes. In fact, TSHR stimulation induced expression of metallothionein isoform 1X (*MT1X*) in human follicular carcinoma cells [25]. *MT1G* (metallothionein 1G) expression was frequently silenced or downregulated in thyroid cancer cell lines and was also significantly decreased in primary thyroid cancer tissues compared with non-malignant thyroid tissues [26]. The segment representation of the transcriptome map could be used to find those chromatin domains including genes less characterized in relation to the thyroid tissue (see Table 1 and TRAM results file available at: http://apollo11.isto.unibo.it/suppl).

At single gene level, the analysis of the most overexpressed genes shows loci which might have a typical thyroid function. These include well known genes and uncharacterized loci whose investigation should be furthered (Table 2). The known gene most expressed is again *TG* (expression value = 8,662.34) (Table 2), however, the maximum value of the expression is presented by the EST cluster Hs.732685 (expression value = 10,763.93) mapping within the known locus *MALAT1* (metastasis associated lung adenocarcinoma transcript 1 (non-protein-coding)) on chr11, as shown by BLASTN analysis. We found that the platform probe (1558678_s_at from GPL570 platform) refers to *MALAT1*, but performing a bioinformatic analysis, we noted that this cluster is mainly characterized by antisense sequences with respect to *MALAT1* orientation. This datum could explain the different expression between the EST cluster Hs.732685 and *MALAT1* (expression values 10,763.92 and 2,180.86, respectively). Due to widespread expression at a high level in human tissues, it is possible that this uncharacterized transcript is a housekeeping gene. This cluster was previously found as overexpressed in hippocampus transcriptome map [18].

The main thyroid function is to produce thyroid hormones in large amounts and this may be reflected by the fact that among the 5 most expressed genes are included *RPL41* (expression value = 5903.29), encoding for ribosomal protein L41, and *EEF1A1* (expression value = 5537.45) encoding for eukaryotic translation elongation factor 1 alpha 1, which is responsible for the enzymatic delivery of aminoacyl tRNAs to the ribosome [27]. Their overexpression could be considered a sign of active protein synthesis. Analysis of EST database for *EEF1A1* showed a very high expression through a variety of human tissues and in agreement with its basic housekeeping function, a very high expression level in cells actively producing proteins in the process of translation is not surprising.

Another highly expressed known gene is *TPT1* (expression value = 5743.83) encoding for the tumor protein, translationally-controlled 1 (TCTP) which is a highly-conserved, cyto-protective protein implicated in many physiological and disease processes, in particular cancer, where it is associated with unfavorable prognosis [28].

Due to the fact that DS, in which the trisomy of chromosome 21 leads to an altered gene expression condition, is characterized by a high prevalence of thyroid dysfunction, in particular congenital hypothyroidism [29], particular attention was paid to the expression of chr21 genes which were already overexpressed in transcriptome maps generated by TRAM when comparing diploid and trisomic blood cells [30]. In our thyroid transcriptome map the most expressed chr21 gene is *TFF3* (expression value = 2726.93). Recently *TFF3* has been described as a gene that could function as an oncogene or oncosuppressor in dependence on the cellular context. In fact, in many types of solid tumors an increase of *TFF3* expression level was observed, while in all thyroid cancers of follicular cell origin its expression was decreased when in normal thyroid it was highly expressed [31]. Other known overexpressed genes on chr21 are *CSTB* (cystatin B) and *SOD1* (superoxide dismutase 1, soluble) (Table 2) encoding, respectively, for a protease inhibitor that has been reported to protect neurons from oxidative stress and for a free superoxide radical destroyer that controls the redox state of the cells. Recent results support the hypothesis of a direct interaction between the two proteins that may be relevant to render cells more responsive to oxidative stress conditions [32]. The Authors suggest a common activation pathway of their transcription. The expression ratio, obtained from TRAM analysis, between the 2 genes is 1.098, consistent with a stoichiometric molecular ratio of 1:1 between proteins that work associated in a molecular complex. It would be interesting to deepen the functions of the overexpressed chr21 genes, to study them in relation to the pathogenesis of DS, and to search for new genes on chr21 (where there are only 273 known genes [33]) in light of the fact that a very small region apparently intergenic appears to be conserved in all subjects diagnosed with DS [34]. The study of pathogenesis of congenital hypothyrodism as well as of other alterations associated to DS will also take advantage of the just released transcriptomic atlas obtained by RNA-Seq method in 15 normal human embryo tissues and organs, among which is included the thyroid [35].

### Differential transcriptome map: Thyroid vs. pool of non-thyroid tissues

To highlight the thyroid-specific gene expression profile, a comparison pool composed of 624 samples derived from 53 different human tissues or organs has been accurately assembled. The differential transcriptome map was obtained by the comparison of the whole thyroid vs. the pool of non-thyroid tissues. The last allowed us to obtain an average value for each gene from non-thyroid tissues.

The differential transcriptome map showed the overexpression of chromatin domains including genes known to be involved in the thyroid function. For example, the first segment on chr8 again includes the known gene *TG*, followed by a segment on chr14 including *NKX2-1*. We also found *IYD* in the third segment on chr6 and *PAX8* in the fourth segment on chr2 (Table 1). All these genes, encoding for thyroid specific transcriptional factors or enzymes, are fundamental in thyroid hormone synthesis [36].

Detailed analysis of the results can be extended to all over- and underexpressed segments (see TRAM results file available at: http://apollo11.isto.unibo.it/suppl). The results proved that, among the overexpressed segments, those including genes with a typical thyroid function prevail, unlike what can be observed among the underexpressed segments as expected by a differential transcriptome map of the thyroid vs. all non-thyroid tissues (Table 1).

Analysis at the single gene level shows a molecular atlas of the thyroid functions and histology structure. Simply observing the list of the first 100 genes (out of a total of 26,750 loci; Additional file 4), ordered according to the decreasing differential expression values, we found genes involved in all phases of the thyroid hormone synthesis. Once again the most overexpressed gene is *TG* encoding for the scaffold for the iodination of tyrosine and the thyroid hormone storage form, followed by *TPO* whose product performs iodination of tyrosine residues in TG and phenoxy-ester formation between pairs of iodinated tyrosines to generate the thyroid hormones. They were followed by the known coding genes *SLC26A7*, *IYD*, *TSHR* and *SLC26A4* (solute carrier family 26 member 4) (Table 3 and Additional file 4). IYD catalyzes the deiodination of mono- and diiodotyrosine and is necessary for iodide salvage [37] and further production of hormones. The presence of abundant TSHRs on thyroid cells is crucial for their response to the main thyroid stimulating factor TSH. SLC26A7 and SLC26A4 belong to an anion exchangers family [38] and in particular, SLC26A4 is known to exchange iodine ions. Also presenting high differential expression values (between 18.20 and 12.54-fold increase respect to non-thyroid tissues), we noted *NKX2-1*, *PAX8*, *FOXE1* and *HHEX* (hematopoietically expressed homeobox) genes that encode for transcription factors essentially for thyroid development and growth as for the adult hormonogenesis through the expression regulation of genes involved in this process. These transcription factors are expressed also in other tissues, but are coexpressed only in the thyroid [39]. Other high differentially expressed genes (differential expression ratio > 7) with known thyroid functions are *DIO1* and *DIO2*, encoding for deiodinases catalyzing the conversion of T_4_ in T_3_, the more active form of thyroid hormone, and *DUOX2* encoding for an enzyme included in a peroxide generating system that is part of the protein complex catalyzing thyroid hormone synthesis together with TPO [40, 41].

We may also consider that the functional importance of some genes is not always linked to their level of expression. It is a classical axiom that thyroid produces thyroid hormones and calcitonin, but how much is the calcitonin gene expressed? In our map, the *CALCA* (calcitonin related polypeptide alpha) gene, encoding the principal form of calcitonin produced by the thyroid, showed an absolute expression value in the thyroid of 218.27 vs. the *TG* expression value of 8,662.34 (ratio = 39.69), when the *CALCA* thyroid value was compared with the mean value obtained from the pool of non-thyroid tissue the differential expression ratio was 3.65-fold increase (*TG* showed a differential expression ratio = 188.22) with a difference between the two genes of 51.57 folds (Additional file 4). It should also be considered that the calcitonin-producing cells are quantitatively much less of the follicular cells (see Background section) and that in any case a differential expression of 3.5 times is significant.

Among chr21 genes, an expression ratio higher than 10 was observed for *TFF3* gene (expression ratio = 16.14), which is again the most overexpressed chr21 gene (Table 3) probably because of its typical function in normal thyroid. It is followed by the uncharacterized EST cluster Hs.129583 (expression ratio = 11.10) (Table 3).

It should be noted here that, when dealing with the lowest gene expression values in our map, they are descriptive of very low or null activity of the gene, but they do not permit the drawing of conclusions about biological information related to specific gene repression in the considered tissue, because the use of a pool of various non-thyroid tissues in our general model might obscure whether a gene is specifically down-regulated in thyroid or is very highly expressed tissue-specifically elsewhere. However, the availability of the data for both thyroid alone (absolute value) and thyroid vs. non-thyroid pool (ratio value) may help in analyzing single situations of interest.

This quantitative representation of gene expression in the human thyroid, obtained without any a priori specific assumption and fully coherent with established biological knowledge about thyroid structure/function, seems to show at the molecular level the classical description of the basic histology of thyroid tissue. This also suggests that other genes with less characterized roles in the thyroid, but with a relevant specific expression in this organ, may have critical functions for thyroid molecular physiopathology. For instance, *SLC26A7*, presently known for its role in the kidney (NCBI Gene entry 115111), turns out here to be the known gene most overexpressed in thyroid in comparison to non-thyroid tissues (67-fold) while simultaneously having to date no thyroid-related function or phenotype described for it (Tables 3 and 5). Interestingly, a syndrome characterized by deafness and thyroid goiter (Pendred syndrome) has been associated to mutations of the related gene *SLC26A4*, also belonging to the same anion transporter gene family and overexpressed in thyroid (Table 5). Uncharacterized EST clusters highly and specifically expressed in thyroid (Table 3) might be associated in the same way to presently unknown functional loci with a relevant role in this organ.

### Gene expression level in the thyroid and association to thyroid mutant phenotypes

The interesting finding of a very high differential expression level in the thyroid respect to non-thyroid tissues for a number of thyroid specific genes encoding for proteins fundamental in thyroid hormone production, led us to verify if there was a correlation between the high differential expression level and the occurrence of a pathological phenotype when these genes are mutated. Even considering only the first 50 most expressed known genes in the thyroid, a statistically significant association (*p* = 0.023) between the entity of the gene differential expression profile (A/B ratio values) in the thyroid vs. non-thyroid tissues and the description of pathological thyroid phenotypes in OMIM when these genes are mutated may be observed (Table 5).

Remarkably, this is a demonstration that reference quantitative expression values for a gene in a tissue could be systematically used as clues to associate a mutation in that gene, or an abnormal quantitative variation in its expression, to an increased probability that the alteration might affect the function of that given human tissue. The map described here could function as a forecasting map providing the possibility to discover new genes with key roles in the thyroid and hence supporting further research for a thyroid disease gene. However, it should be noted that also mutations of genes expressed at low level, as those for some transcription factors or regulative RNAs, could have a strong impact on the phenotype.

### Sex-biased gene expression in the human thyroid

We performed a study of sex-biased gene expression in the whole thyroid gland to investigate whether gender has a significant influence on the thyroid gene expression profile observed in our analysis. For this purpose an additional differential transcriptome map to investigate specifically sex-biased gene expression patterns was obtained.

Interestingly, when sorting by Pool A.1 (male thyroid) expression values, we noted that the more expressed genes have a similar expression pattern in both sexes with differential ratios close to 1 (Additional file 6). These results are an additional confirmation of the reliability of the data produced by our map and support a high differential expression mainly for a small percentage of genes that are known to have a different expression between males and females, most of them sex-linked, while genes important for the thyroid function remain similarly expressed in both sexes (Fig. 2). Melé et al. [16] recently demonstrated that the variation in gene expression is greater among tissues (47%) than among individuals (4%).

### Experimental validation of the thyroid transcriptome map

To prove the reliability of the thyroid transcriptome map generated by TRAM software, we performed an experimental validation of the obtained data. The excellent correlation between the expected and observed data (*r* = 0.93, *p*-value < 0.0001) (Fig. 3) allows us to consider all other intervening values among the points we selected as bona fide values, thus for the first time providing a reliable and quantitative representation of the reference expression values for 27,275 mapped transcripts and 13,002 uncharacterized (unmapped) transcripts of the whole normal human thyroid. These data suggest that our systematic transcriptome map, while highlighting interesting aspects of the structure and function of the normal human thyroid, may be a useful tool as a quantitative reference for gene expression studies related to this gland, as a whole, in a physiologically normal condition.

The quantitative results typically provided by the TRAM approach compare favorably with the ones provided by other available tools able to extract information about gene expression levels from published datasets, in that the advanced probe-to-locus assignment process maximizes the number of transcripts for which an expression value is obtained, while the powerful algorithm of data integration from diverse experimental platforms attenuates systematic biases associated to individual platforms. We have previously shown, in the case of brain transcriptome map, that TRAM map offers specific advantages over a specific brain gene expression atlas [12]. To simply but explicatively compare the data presented here for the human thyroid with the ones provided by the EBI Gene Expression Atlas [42] (https://www.ebi.ac.uk/gxa/home), we have extracted from this browser numerical values for the expression level of the 16 genes we have successfully measured by RT-PCR (Additional file 8) by selecting “*Homo sapiens*” as organism and “Thyroid gland” as tissue, then considering the first two proposed datasets, “GTEx” [43] and “Illumina Body Map” (both RNA-Seq based and normalized via RPKM (Reads Per Kilobase Million) method). Correlation coefficient with the in vitro validated values was 0.70 and 0.59, respectively, compared to 0.93 observed with TRAM results (Additional file 8). This fits better at the cost of additional manual curation in the initial steps of the set-up of a TRAM analysis, not needed for the atlases automatically maintained in the web environment; analogous results were obtained by searching BioGPS [44] (data not shown). In the case of TRAM, values are less correlated to the experimentally determined measure while approaching the lower limit of detection, as expected due to the hybridization-based nature of the microarray platforms.

It is worthwhile to finally note that this effective and validated quantitative approach in determining reference expression values for the loci expressed in a transcriptome extends to the ability of TRAM to provide a chromosomal region-based analysis by searching for statistically significant over−/underexpression of genomic segments, not based on the simple enrichment in the segment of over−/underexpressed genes contained in the segment, but on the actual determination of an expression value for the whole genomic segment derived by summarizing the quantitative expression values of the genes contained in it.

### Search for reference genes to study gene expression in the human thyroid

The availability of the systematic and detailed expression map presented here for the thyroid represented an excellent occasion to investigate, among many features of the transcriptome, the suitability of individual genes as the best stable reference genes for data normalization in gene expression studies. This is because they fulfill criteria including a widely diffused, constant and high expression [45].

The best gene at behaving like a housekeeping gene in the thyroid is *ACTG1*, encoding for actin gamma 1, a cytoplasmic actin found in non-muscle cells as a cytoskeletal component (Table 4). The other form of non-muscle cytoskeletal actin is *ACTB* (actin beta). Weber et al. showed that, between 6 typical reference genes, *ACTB* was the most stable and suitable as housekeeping in normal human thyroid and goiter tissues [46]. However, in that study *ACTG1* was not investigated. The gene for the ribosomal protein RPLP0 also presents positively fulfilling the combination of high expression level, low SD among samples and high number of measured data points across multiple samples (Table 4).

## Conclusions

While our quantitative global reference portrait of gene expression in the normal human thyroid shows very high expression of genes known to exert basilar actions for the thyroid functions, in particular when comparing thyroid and non-thyroid tissues, the identification of many other genes or uncharacterized transcripts differentially expressed in the thyroid opens the way for the identification of genetic determinants related to the thyroid physiology.

The experimental in vitro validation as well as the remarkable correlation between the level of transcription of a gene and its probability to be associated, when mutated, to a genetic thyroid disease further supports the possible usefulness of the human thyroid transcriptome map as a reference for molecular studies of the physiology and pathology of this organ. For example, the analysis of chr21 gene quantitative expression levels could be used for successive studies of differential expression between normal thyroid tissue vs. trisomy 21 thyroid tissue, inferring gene behavior in an affected condition.

## Methods

### Database search and selection

A systematic search in gene expression data repositories for any single sample available listing gene expression values for the human thyroid and non-thyroid tissues from subjects explicitly referred to as “healthy” or “normal” was conducted exactly as previously described [47], except than the search term “thyroid” was used instead of “heart”. The criteria for inclusion or exclusion in the analysis of each retrieved dataset also were as previously described [47].

### TRAM analysis

TRAM software [17] allows the import of gene expression data recorded in the GEO and ArrayExpress databases or in a custom source in tab-delimited text format whether the data are referred to microarry, RNA-Seq or proteomic platforms for the gene product expression studies. It also allows the integration of all data by decoding probe set identifiers to gene symbols via UniGene data parsing [48], normalizing data from multiple platforms using intra-sample and inter-sample normalization (scaled quantile normalization) [49], creating a graphical representation of gene expression profiles along the chromosomes and determining the statistical significance of differential expression of chromosomal segments in comparison with the other segments in the biological condition studied and in comparison with values calculated for the corresponding segments in the second biological condition when two conditions A and B are compared. The statistical analysis used by TRAM to this aim is hypergeometric distribution, a recognised algorithm able to test the probability ‘p’ that colocalization of over−/underexpressed genes within the same chromosomal segment may be due to chance [17, 50]. A graphical representation of the overall TRAM software workflow is provided in Fig. 1.

The value for each locus, in each biological condition, is the mean value of all available values for that locus. The genome wide gene expression median value was used in order to determine percentiles of expression for each gene.

Using the “Map” mode graphical representation we searched for over−/underexpressed genome segments which have a window size of 500,000 bp (base pairs) and a shift of 250,000 bp. The expression value for each genomic segment is the mean of the expression values of the loci included in that segment. A segment is defined over−/underexpressed if it has an expression value which is significantly different between two conditions analyzed, and contains at least three individually over−/underexpressed genes, e.g. genes which have expression values within the highest or the lowest 2.5th percentile.

We created two directories (folders) for each source which contain all sample datasets selected for the study. To compare whole thyroid samples with a pool of non-thyroid tissues, we collected the first in a folder named Pool A and the second in a folder named Pool B. In addition, the thyroid samples for which the sex of the sample donor was available were used to provide a differential transcriptome map between male and female thyroids: Male thyroid (Pool A.1) and Female thyroid (Pool A.2), not including samples deriving from mixed male/female tissues (Additional file 1). The comparisons allowed us to analyze differential maps using the ratio of the mean expression values for each locus in addition to the maps related to each single type of sample.

Significance of the over−/underexpression for single genes was determined by running TRAM in “Map” mode and lowering the segment window to 12,500 bp and the minimum number of over−/underexpressed genes in that window to 1. This window size is lower than 20% of the 67-kb mean size of a human protein-coding gene (as determined by searching the recent GeneBase database [51]: mean of gene size from 17,958 “reviewed” or “validated” entries in the NCBI Gene April 2015 annotation release), so the significant over−/underexpression of a segment almost always corresponds to that of a gene. When the segment window contains more than one gene, the significance is maintained if the expression value of the over−/underexpressed gene prevails over the others.

Sample expression values equal to or lower than “0” (≤ 0) will be thresholded by TRAM [47] to 95% of the minimum positive value present in that sample, in order to obtain meaningful numbers when dividing “Samples Pool A” values by “Sample Pool B” values. Assuming that in these cases an expression level is too low to be detected under the used experimental conditions, this transformation still allows a ratio between values in the Pool A and values in the Pool B to be obtained, which is useful to highlight differential gene expression.

### Housekeeping gene search

We determined the genes that behave like housekeeping genes, in that they are mainly involved in fundamental cellular functions and tend to be constantly expressed at a high level, typically, but not necessarily, in a consistent way across many different tissues/organs [52, 53]. A search of housekeeping genes best suitable for the study of human thyroid was performed in the transcriptome map of human thyroid created as described above (Pool A) using the following parameters in combination: expression value > 100 in order to select genes expressed above the mean value (that is posed equal to 100) and so at an appreciable level; data points number (where a data point is a measure of the expression value for a gene obtained from a single spot on the array) ≥ of half the number of samples of the map in order to select commonly expressed genes (≥ 18); SD, expressed as a percentage of the mean value, ≤ 30 in order to identify genes with a low expression variation among different thyroid samples.

### Gene expression level in the thyroid and association to thyroid phenotypes

The availability of a measured ratio of the expression level of a gene in the thyroid vs. non-thyroid tissues provides means to formally test the hypothesis that the mutation in a gene with a typical high expression level in the thyroid, in comparison with non-thyroid tissues, is most likely that it will result in a pathologic thyroid-related phenotype. For this purpose, for each of the first 50 known genes most expressed in thyroid respect to the mean of non-thyroid tissues (ratio A/B ≥ 5.49), the certain association to a pathological thyroid phenotype was recorded by manual searching of the OMIM database [54]. Following sorting of the genes by their differential expression value in descending order, Fisher’s exact test was used to compare the first 25 genes against the following 25 genes in thyroid vs. non-thyroid tissues differential transcriptome map to test the significance of the association of a high ratio expression value to the OMIM description of a pathological thyroid phenotype when they are mutated. In this analysis we have considered only genes with the following parameters: data points number ≥ 5 both in Pool A and Pool B; SD, expressed as a percentage of the mean value, < 200% when expression value was > 50 (high SD is tolerated in the low range of values due to a tendency of imprecision when approaching the low limit of detection).

### In vitro validation of the thyroid transcriptome map

In order to obtain experimental confirmation of the meta-analysis derived map, we randomly selected a group of genes with expression values covering the whole range of the expression magnitude order as calculated by TRAM. In the validation set, thyroid-specific genes (*TPO*, *IYD*, *TSHR*) and genes which are not commonly considered to have a specific thyroid function (*RPL41*, *B2M*, *SERPINF1*, *BACE2*, *NTNG1*, *NPTX1*, *TBX18*) were included. The genes are known and characterized, also verifying that they are not included among the genes with a known incomplete determination of their 5′ coding sequence [55].

Complementary DNA (cDNA) templates were obtained from reverse transcription of commercial total RNA extracted from human normal thyroids pooled from 64 male/female Caucasians, aged between 15 and 61 years with sudden death as the reported cause of death while no further RNA source information is available from the provider (Clontech, Mountain View, CA). The reverse transcription was performed according to [47].

Primer pairs were designed with “Amplify 3” software [56] following standard criteria [57]. They are designed on different exons, except for the *ACTB* gene, to specifically recognize expressed sequences and to bind to regions common to all isoforms of the same gene since microarray probe sequences are complementary to sequences common of the known isoforms of the same gene. These constraints caused the amplicon length variation between 99 and 247 bp (Additional file 7). Primers for *ACTB* gene are designed on the same exon to avoid unspecific products and to amplify the same trait recognized by the probes used in microarray platforms. A control PCR reaction was performed using the commercial RNA directly as a template to verify a potential contaminating DNA amplification by the commercial RNA sample.

Real-Time PCR assays were performed in triplicate, using the CFX96 instrument (Bio-Rad Laboratories, Hercules, CA). The reactions were performed in a total volume of 20 μL using Sybr Select Master Mix 2× for CFX (Applied Biosystem, by Life Technologies) according to manifacturer instructions. Cycling parameters were: 2 min at 50 °C (UDG activation), 2 min at 95 °C (AmpliTaq Fast DNA Polymerase UP activation), 40 cycles of 15 s at 95 °C (denature) and of 1 min at 60 °C (anneal and extend). A melting step needs to be performed to assay amplification specificity. This step consisted of an increase in temperature of 0.5 °C/s from 65 °C to 95 °C.

For each gene we used the primer pair that gave between 90 and 110% efficiency. We used the 2^∆Ct^ (delta cycle threshold) method, a variation of the Livak method [58], that uses the difference between reference and target gene Ct values for each sample to do a relative quantification normalized to a reference gene (Observed Ratio_(reference/target)_ = 2^Ct(reference) – Ct(target)^). We set the gene with an intermediate expression value and a low standard deviation (SD, expressed as percentage of the mean value) in TRAM analysis as reference gene. The ratio among the transcriptome map expression values was calculated by dividing each expression value of the target gene by the expression value of the reference (expected ratio). Then we compared this value with the observed ratio and we examined the relationship between these two variables through bivariate statistical analysis using JMP 5.1 software (SA Institute, Campus Drive Cary, NC).

## Additional files

Additional file 1: Table S1. Samples selected for the meta-analysis of gene expression profiles in thyroid (Pool A), male thyroid (Pool A.1), female thyroid (Pool A.2). All samples are related to human healthy individuals. (PDF 110 kb)

Additional file 2: Table S2. Samples selected for the meta-analysis of gene expression profiles in pool of non-thyroid tissues (Pool B). (PDF 628 kb)

Additional file 3: Table S3. List of 27,275 loci of the whole normal thyroid transcriptome map. Loci are sorted in decreasing order of expression value. N/A = not available in the Gene database (http://www.ncbi.nlm.nih.gov/gene) when the analysis was performed. (XLS 3372 kb)

Additional file 4: Table S4. List of 26,750 loci of the differential transcriptome map between Whole normal thyroid (Pool A) and Pool of non-thyroid tissues (Pool B). Loci were sorted in descending order of expression ratio (Ratio A/B). N/A: not available in the Gene database (http://www.ncbi.nlm.nih.gov/gene) when the analysis was performed. (XLS 4948 kb)

Additional file 5: Table S5. List of 25,954 loci of the differential transcriptome map between Male thyroid (Pool A.1) and Female thyroid (Pool A.2). Loci were sorted in descending order of expression ratio (Ratio A.1/A.2). N/A: not available in the Gene database (http://www.ncbi.nlm.nih.gov/gene) when the analysis was performed. (XLS 4795 kb)

Additional file 6: Table S6. List of 25,954 loci of the differential transcriptome map between Male thyroid (Pool A.1) and Female thyroid (Pool A.2). Loci were sorted in descending order of A.1 expression values. N/A: not available in the Gene database (http://www.ncbi.nlm.nih.gov/gene) when the analysis was performed. (XLS 5255 kb)

Additional file 7: Table S7. PCR primer pairs used to validate thyroid TRAM map. (PDF 96 kb)

Additional file 8: Table S8. Comparison of the data presented here for the expression level of the 16 genes of the human thyroid transcriptome map we have successfully measured by RT-PCR with the ones provided by the EBI Gene Expression Atlas, considering the first two proposed datasets, “GTEx” and “Illumina Body Map” (both RNA-Seq based and normalized via RPKM (Reads Per Kilobase Million) method). (XLS 56 kb)

## Abbreviations

cDNA: complementary DNA
DS: Down syndrome
EBI: European bioinformatics institute
EST: expressed sequence tag
GEO: Gene expression omnibus
NCBI: National center for biotechnology information
OMIM: Online mendelian inheritance in man
RNA-Seq: RNA sequencing
RT-PCR: reverse transcription - polymerase chain reaction
SD: standard deviation
TRAM: Transcriptome mapper (software)
