# Supplementary material for: A molecular view of the normal human thyroid structure and function reconstructed from its reference transcriptome map
Source: BMC Genomics. 2017 Sep 18;18:739. doi: 10.1186/s12864-017-4049-z (PMC5604164; doi:10.1186/s12864-017-4049-z)
Supplement: Supplementary file 1 — Samples selected for the meta-analysis of gene expression profiles in thyroid (Pool A), male thyroid (Pool A.1), female thyroid (Pool A.2). All samples are related to human healthy individuals. (PDF 110 kb) [file 12864_2017_4049_MOESM1_ESM.pdf]

Additional file 1: Table S1. Samples selected for the meta-analysis of gene expression profiles in thyroid (Pool A), male thyroid (Pool A.1), female thyroid (Pool A.2). All samples are related to human healthy individuals.

Study ID	Sample ID	Age	Sex	Sample type	Sample collection	Post-mortem interval	Platform	Microarray	Spots	References
**Pool A - Thyroid** (27,275 mapped loci following analysis by TRAM) **(n=35)**										
A1 - A2<br>n=2	NO1 - NO2*<br>(E-MEXP-2442)[a]	N/A<br>(adult)	2 F	Whole thyroid	Institutions participating in the study	N/A	A-AFFY-44*<br>(GPL570)	Affymetrix U133 Plus 2.0	54,675	(Borup et al., 2010)
A3 - A4<br>n=2	GSM2836<br>GSM2848	N/A	N/A	"	Commercial human thyroid RNA	N/A	GPL91	Affymetrix U95A	12,626	(Su et al., 2002)
A5 - A6<br>n=2	GSM18802 - 03	N/A	M/F mix	"	Commercial and Institutions participating in the human thyroid RNA study	N/A	GPL1074	GNF1H	22,639	(Su et al., 2004)
A7 - A8<br>n=2	GSM18961 - 62	N/A	M/F mix	"	"	N/A	GPL96	Affymetrix U133A	22,283	(Su et al., 2004)
A9<br>n=1	GSM44685	N/A	M/F mix	"	Commercial human thyroid RNA	N/A	GPL96	Affymetrix U133A	22,283	(Ge et al., 2005)
A10...A13<br>n=4	GSM80864...67	25y, 38y, 39y, 30y	3 M<br>1 F	"	Institutions participating in the study	0 h - 8.5 h	GPL570	Affimetrix U133 Plus 2.0	54,675	(Roth et al., 2006)
A14...A18<br>n=5	GSM175954<br>GSM176418...21	N/A	1 M/F mix<br>3 M<br>1 F	"	"	N/A	GPL570	Affimetrix U133 Plus 2.0	54,675	N/A
A19...A21<br>n=3	GSM194528...30	N/A	N/A	"	Commercial human thyroid RNA	N/A	GPL2986	ABI Human Genome Survey Microarray	32,878	(Dezso et al., 2008)

								Version 2		
A22 - A23 n=2	GSM201392 - 93	N/A	M/F mix	"	"	N/A	GPL1291	Hitachisoft AceGene Human Oligo Chip 30K 1 Chip Version	30,336	(Kato et al., 2008)
A24...A31 n=8	GSM422030...37	68y	8 F	"	"	N/A	GPL6254	Phalanx Human OneArray	30,968	(Abu-Khudir et al., 2010)
A32...A35 n=4	GSM671268...71	N/A	N/A	"	Institutions participating in the study	N/A	GPL96	Affymetrix U133A	22,283	(Giordano et al., 2006; Giordano et al., 2005)
**Pool A.1 - Male thyroid (**25,954 mapped loci following analysis by TRAM**)** **n= 6**										
A1...A3 n=3	GSM80864 - 65 - 67	25y, 38y, 30y	3 M	Whole thyroid	Institutions participating in the study	0 h - 8.5 h	GPL570	Affimetrix U133 Plus 2.0	54,675	(Roth et al., 2006)
A4...A6 n=3	GSM176418 - 19 - 21	N/A	3 M	"	"	N/A	GPL570	Affimetrix U133 Plus 2.0	54,675	N/A
**Pool A.2 - Female thyroid (**26,392 mapped loci following analysis by TRAM**)** **n= 12**										
A1...A2 n=2	NO1 - NO2* (E-MEXP-2442)[a]	N/A (adult)	2 F	Whole thyroid	Institutions participating in the study	N/A	A-AFFY-44* (GPL570)	Affimetrix U133 Plus 2.0	54,675	(Borup et al., 2010)
A3 n=1	GSM80866	39y	1 F	"	"	0 h - 8.5 h	GPL570	Affimetrix U133 Plus 2.0	54,675	(Roth et al., 2006)
A4 n=1	GSM176420	N/A	1 F	"	"	N/A	GPL570	Affimetrix U133 Plus 2.0	54,675	N/A
A5 - A12 n=8	GSM422030...37	68y	8 F	"	Commercial human thyroid RNA	N/A	GPL6254	Phalanx Human OneArray	30,968	(Abu-Khudir et al., 2010)

From the left to right: our study ID; GEO (Gene Expression Omnibus) sample ID; sex, when it is available; sample type; sample collection; post-mortem interval (PMI) before freezing; GEO platform name; platform type; platform spot number and GEO experiment reference. All sample IDs

and platforms IDs marked with * are related to ArrayExpress database. [a]This is the ArrayExpress experiment number provided because the only sample number does not allow to identify data source. N/A: not available.

REFERENCES

Abu-Khudir, R., Paquette, J., Lefort, A., Libert, F., Chanoine, J.P., Vassart, G., Deladoey, J., 2010. Transcriptome, methylome and genomic variations analysis of ectopic thyroid glands. PLoS One 5, e13420.

Borup, R., Rossing, M., Henao, R., Yamamoto, Y., Krogdahl, A., Godballe, C., Winther, O., Kiss, K., Christensen, L., Hogdall, E. et al., 2010. Molecular signatures of thyroid follicular neoplasia. Endocr. Relat. Cancer 17, 691-708.

Dezso, Z., Nikolsky, Y., Sviridov, E., Shi, W., Serebriyskaya, T., Dosymbekov, D., Bugrim, A., Rakhmatulin, E., Brennan, R.J., Guryanov, A. et al., 2008. A comprehensive functional analysis of tissue specificity of human gene expression. BMC Biol. 6, 49.

Ge, X., Yamamoto, S., Tsutsumi, S., Midorikawa, Y., Ihara, S., Wang, S.M., Aburatani, H., 2005. Interpreting expression profiles of cancers by genome-wide survey of breadth of expression in normal tissues. Genomics 86, 127-141.

Giordano, T.J., Au, A.Y., Kuick, R., Thomas, D.G., Rhodes, D.R., Wilhelm, K.G., Jr., Vinco, M., Misek, D.E., Sanders, D., Zhu, Z. et al., 2006. Delineation, functional validation, and bioinformatic evaluation of gene expression in thyroid follicular carcinomas with the PAX8-PPARG translocation. Clin. Cancer Res. 12, 1983-1993.

Giordano, T.J., Kuick, R., Thomas, D.G., Misek, D.E., Vinco, M., Sanders, D., Zhu, Z., Ciampi, R., Roh, M., Shedden, K. et al., 2005. Molecular classification of papillary thyroid carcinoma: distinct BRAF, RAS, and RET/PTC mutation-specific gene expression profiles discovered by DNA microarray analysis. Oncogene 24, 6646-6656.

Kato, M., Miya, F., Kanemura, Y., Tanaka, T., Nakamura, Y., Tsunoda, T., 2008. Recombination rates of genes expressed in human tissues. Hum. Mol. Genet. 17, 577-586.

Roth, R.B., Hevezi, P., Lee, J., Willhite, D., Lechner, S.M., Foster, A.C., Zlotnik, A., 2006. Gene expression analyses reveal molecular relationships among 20 regions of the human CNS. Neurogenetics 7, 67-80.

Su, A.I., Cooke, M.P., Ching, K.A., Hakak, Y., Walker, J.R., Wiltshire, T., Orth, A.P., Vega, R.G., Sapinoso, L.M., Moqrich, A. et al., 2002. Large-scale analysis of the human and mouse transcriptomes. Proc. Natl. Acad. Sci. U. S. A. 99, 4465-4470.

Su, A.I., Wiltshire, T., Batalov, S., Lapp, H., Ching, K.A., Block, D., Zhang, J., Soden, R., Hayakawa, M., Kreiman, G. et al., 2004. A gene atlas of the mouse and human protein-encoding transcriptomes. Proc. Natl. Acad. Sci. U. S. A. 101, 6062-6067.
