# Supplementary material for: A molecular view of the normal human thyroid structure and function reconstructed from its reference transcriptome map
Source: BMC Genomics. 2017 Sep 18;18:739. doi: 10.1186/s12864-017-4049-z (PMC5604164; doi:10.1186/s12864-017-4049-z)
Supplement: Supplementary file 2 — Samples selected for the meta-analysis of gene expression profiles in pool of non-thyroid tissues (Pool B). (PDF 628 kb) [file 12864_2017_4049_MOESM2_ESM.pdf]

Additional file 2: Table S2. Samples selected for the meta-analysis of gene expression profiles in pool of non-thyroid tissues (Pool B).

Study ID	SAMPLE ID	Sex	Sample Type	Sample collection	Post-mortem interval	Platform	Microarray	Spot	Ref.
**B1**	GSM80561	M	ADIPOSE TISSUE (Omental)	Institution participating in the study	8.5 h	GPL570	Affimetrix U133 Plus 2.0	54,675	[1]
**B2**	GSM80562	"	"	"	"	"	"	"	"
**B3**	GSM80563	F	"	"	"	"	"	"	"
**B4**	GSM80564	M	"	"	"	"	"	"	"
**B5**	GSM80580	F	ADIPOSE TISSUE	"	"	"	"	"	"
**B6**	GSM80583	M	"	"	"	"	"	"	"
**B7**	GSM80584	"	ADIPOSE TISSUE (Subcutaneous)	"	"	"	"	"	"
**B8**	GSM80588	"	ADIPOSE TISSUE	"	"	"	"	"	"
**B9**	GSM80589	"	ADIPOSE TISSUE (Subcutaneous)	"	"	"	"	"	"
**B10**	GSM80590	"	"	"	"	"	"	"	"
**B11**	GSM175834	M/F mix	ADIPOSE TISSUE (Omental)	Commercial human total RNA from donors	N/A	"	"	"	N/A
**B12**	GSM175835	M	"	"	"	"	"	"	"
**B13**	GSM175836	F	"	"	"	"	"	"	"
**B14**	GSM175837	M	"	"	"	"	"	"	"
**B15**	GSM175994	F	ADIPOSE TISSUE	"	"	"	"	"	"
**B16**	GSM176027	M	"	"	"	"	"	"	"
**B17**	GSM176028	"	ADIPOSE TISSUE (Subcutaneous)	"	"	"	"	"	"
**B18**	GSM176118	"	ADIPOSE TISSUE	"	"	"	"	"	"
**B19**	GSM176119	"	ADIPOSE TISSUE (Subcutaneous)	"	"	"	"	"	"
**B20**	GSM176121	"	"	"	"	"	"	"	"
**B21**	GSM2827	N/A	ADRENAL GLAND	"	"	GPL91	Affymetrix U95A	12,626	[2]
**B22**	GSM2842	"	"	"	"	"	"	"	"
**B23**	GSM18788	"	"	"	"	GPL1074	GNF1H	22,639	[3]
**B24**	GSM18789	"	"	"	"	"	"	"	"
**B25**	GSM18947	"	"	"	"	GPL96	Affimetrix U133A	22,283	[3]
**B26**	GSM18948	"	"	"	"	"	"	"	"
**B27**	GSM39896	"	"	Institution participating in the study	30 min	GPL1823	SHBW	43,008	[4]
**B28**	GSM39906	"	"	"	"	"	"	"	"
**B29**	GSM39961	"	"	"	"	GPL1825	SHBA	44,160	[4]

**B30**	GSM39986	"	"	"	"	"	"	"	"
**B31**	GSM176016	M	AORTA	Commercial human total RNA from donors	N/A	GPL570	Affimetrix U133 Plus 2.0	54,675	N/A
**B32**	GSM176112	F	"	"	"	"	"	"	"
**B33**	GSM176263	M	"	"	"	"	"	"	"
**B34**	GSM176264	F	"	"	"	"	"	"	"
**B35**	GSM18840	N/A	APPENDIX	"	"	GPL1074	GNF1H	22,639	[3]
**B36**	GSM18841	"	"	"	"	"	"	"	"
**B37**	GSM18999	"	"	"	"	GPL96	Affimetrix U133A	22,283	[3]
**B38**	GSM19000	"	"	"	"	"	"	"	"
**B39**	GSM39895	N/A	BLADDER	Institution participating in the study	30 min	GPL1823	SHBW	43,008	[4]
**B40**	GSM39918	"	"	"	"	"	"	"	"
**B41**	GSM44682	"	"	Commercial human total RNA from donors	N/A	GPL96	Affimetrix U133A	22,283	[5]
**B42**	GSM18708	N/A	BLOOD, WHOLE	"	"	GPL1074	GNF1H	22,639	[3]
**B43**	GSM18709	"	"	"	"	"	"	"	"
**B44**	GSM18867	"	"	"	"	GPL96	Affimetrix U133A	22,283	[3]
**B45**	GSM18868	"	"	"	"	"	"	"	"
**B46**	GSM18750	N/A	BONE MARROW	"	"	GPL1074	GNF1H	22,639	[3]
**B47**	GSM18751	"	"	"	"	"	"	"	"
**B48**	GSM18909	"	"	"	"	GPL96	Affimetrix U133A	22,283	[3]
**B49**	GSM18910	"	"	"	"	"	"	"	"
**B50**	GSM44693	"	"	"	"	"	"	"	[5]
**B51**	GSM80576	M	"	Institution participating in the study	8.5 h	GPL570	Affimetrix U133 Plus 2.0	54,675	[1]
**B52**	GSM80577	"	"	"	"	"	"	"	"
**B53**	GSM80602	F	"	"	"	"	"	"	"
**B54**	GSM80603	"	"	"	"	"	"	"	"
**B55**	GSM80604	"	"	"	"	"	"	"	"
**B56**	GSM175906	M/F mix	"	Commercial human total RNA from donors	N/A	"	"	"	N/A
**B57**	GSM175951	N/A	"	"	"	"	"	"	"
**B58**	GSM175974	M	"	"	"	"	"	"	"
**B59**	GSM175975	"	"	"	"	"	"	"	"
**B60**	GSM176300	F	"	"	"	"	"	"	"
**B61**	GSM2828	N/A	BRAIN	"	"	GPL91	Affymetrix U95A	12,626	[2]
**B62**	GSM2837	"	"	"	"	"	"	"	"

**B63**	GSM18762	M/F mix	"	"	"	GPL1074	GNF1H	22,639	[3]
**B64**	GSM18763	"	"	"	"	"	"	"	"
**B65**	GSM18921	"	"	"	"	GPL96	Affimetrix U133A	22,283	"
**B66**	GSM18922	"	"	"	"	"	"	"	"
**B67**	GSM44690	N/A	"	"	"	"	"	"	[5]
**B68**	GSM52556	"	"	Institutions participating in the study	"	"	"	"	[6, 7]
**B69**	GSM88577	"	"	Commercial human total RNA from donors	"	GPL1426	ABI Version 1	33,202	N/A
**B70**	GSM88578	"	"	"	"	"	"	"	"
**B71**	GSM88579	"	"	"	"	"	"	"	"
**B72**	GSM88580	"	"	"	"	"	"	"	"
**B73**	GSM89144	"	"	N/A	"	GPL1708	Agilent-012391 G4112A	44,290	"
**B74**	GSM89145	"	"	"	"	"	"	"	"
**B75**	GSM89146	"	"	"	"	"	"	"	"
**B76**	GSM89147	"	"	"	"	"	"	"	"
**B77**	GSM339552	"	"	Institutions participating in the study	"	GPL96	Affimetrix U133A	22,283	[8]
**B78**	GSM339553	"	"	"	"	"	"	"	"
**B79**	GSM339554	"	"	"	"	"	"	"	"
**B80**	GSM339555	"	"	"	"	"	"	"	"
**B81**	GSM339556	"	"	"	"	"	"	"	"
**B82**	GSM790820	M	"	Commercial human total RNA from donors	"	GPL4133	Agilent-014850 G4112F	45,220	[9]
**B83**	GSM790829	"	"	"	"	"	"	"	"
**B84**	GSM790838	"	"	"	"	"	"	"	"
**B85**	GSM175792	F	BREAST	"	"	GPL570	Affimetrix U133 Plus 2.0	54,675	N/A
**B86**	GSM175795	"	"	"	"	"	"	"	"
**B87**	GSM790821	"	"	"	"	GPL4133	Agilent-014850 G4112F	45,220	[9]
**B88**	GSM790830	"	"	"	"	"	"	"	"
**B89**	GSM790839	"	"	"	"	"	"	"	"
**B90**	GSM80578	N/A	BRONCHUS	Institution participating in the study	8.5 h	GPL570	Affimetrix U133 Plus 2.0	54,675	[1]
**B91**	GSM80579	"	"	"	"	"	"	"	"
**B92**	GSM80582	"	"	"	"	"	"	"	"
**B93**	GSM175978	M	"	Commercial human total RNA from donors	N/A	"	"	"	N/A
**B94**	GSM175979	F	"	"	"	"	"	"	"
**B95**	GSM176022	M	"	"	"	"	"	"	"

**B96**	GSM18844	N/A	CILIARY GANGLION	"	"	GPL1074	GNF1H	22,639	[3]
**B97**	GSM18845	"	"	"	"	"	"	"	"
**B98**	GSM19003	"	"	"	"	GPL96	Affimetrix U133A	22,283	[3]
**B99**	GSM19004	"	"	"	"	"	"	"	"
**B100**	GSM39943	"	COLON	Institution participating in the study	30 min	GPL1824	SHCN	43,008	[4]
**B101**	GSM44680	"	"	Commercial human total RNA from donors	"	GPL96	Affimetrix U133A	22,283	[5]
**B102**	GSM52558	N/A	"	N/A	"	"	"	"	[6, 7]
**B103**	GSM175905	"	"	Commercial human total RNA from donors	N/A	GPL570	Affimetrix U133 Plus 2.0	54,675	N/A
**B104**	GSM774045	"	"	N/A	"	GPL96	Affimetrix U133A	22,283	[10]
**B105**	GSM774046	"	"	"	"	"	"	"	"
**B106**	GSM52564	N/A	CONNECTIVE TISSUE	"	"	"	"	"	[6, 7]
**B107**	GSM27727	N/A	DENTAL PULP	Institution participating in the study	immediately post-extraction	"	"	"	[11]
**B108**	GSM27728	"	"	"	"	"	"	"	"
**B109**	GSM2888	N/A	DORSAL ROOT GANGLIA	Commercial human total RNA from donors	N/A	GPL91	Affymetrix U95A	12,626	[2]
**B110**	GSM2889	"	"	"	"	"	"	"	"
**B111**	GSM18850	"	"	"	"	GPL1074	GNF1H	22,639	[3]
**B112**	GSM18851	"	"	"	"	"	"	"	"
**B113**	GSM19009	"	"	"	"	GPL96	Affimetrix U133A	22,283	"
**B114**	GSM19010	"	"	"	"	"	"	"	"
**B115**	GSM80611	M	"	Institution participating in the study	8.5 h	GPL570	Affimetrix U133 Plus 2.0	54,675	[1]
**B116**	GSM80612	"	"	"	"	"	"	"	"
**B117**	GSM80613	F	"	"	"	"	"	"	"
**B118**	GSM80614	M	"	"	"	"	"	"	"
**B119**	GSM80629	"	"	"	"	"	"	"	"
**B120**	GSM80630	F	"	"	"	"	"	"	"
**B121**	GSM80648	"	"	"	"	"	"	"	"
**B122**	GSM80649	"	"	"	"	"	"	"	"
**B123**	GSM175825	M	"	Commercial human total RNA from donors	N/A	"	"	"	N/A
**B124**	GSM175826	"	"	"	"	"	"	"	"
**B125**	GSM175827	F	"	"	"	"	"	"	"
**B126**	GSM175828	M	"	"	"	"	"	"	"
**B127**	GSM176062	"	"	"	"	"	"	"	"

**B128**	GSM176075	F	"	"	"	"	"	"	"
**B129**	GSM176218	"	"	"	"	"	"	"	"
**B130**	GSM176219	"	"	"	"	"	"	"	"
**B131**	GSM39966	N/A	ESOPHAGUS	Institution participating in the study	30 min	GPL1825	SHBA	44,160	[4]
**B132**	GSM39978	"	"	"	"	"	"	"	"
**B133**	GSM39979	"	"	"	"	"	"	"	"
**B134**	GSM80694	M	"	"	8.5 h	GPL570	Affimetrix U133 Plus 2.0	54,675	[1]
**B135**	GSM80695	"	"	"	"	"	"	"	"
**B136**	GSM80696	F	"	"	"	"	"	"	"
**B137**	GSM80697	M	"	"	"	"	"	"	"
**B138**	GSM176389	"	"	Commercial human total RNA from donors	N/A	"	"	"	N/A
**B139**	GSM176390	"	"	"	"	"	"	"	"
**B140**	GSM176391	F	"	"	"	"	"	"	"
**B141**	GSM176392	M	"	"	"	"	"	"	"
**B142**	GSM39962	N/A	FALLOPIAN TUBE	Institution participating in the study	30 min	GPL1825	SHBA	44,160	[4]
**B143**	GSM39964	"	"	"	"	"	"	"	"
**B144**	GSM39991	"	"	"	"	GPL1826	SHDP	43,104	
**B145**	GSM40000	"	"	"	"	"	"	"	"
**B146**	GSM176032	F	"	Commercial human total RNA from donors	N/A	GPL570	Affimetrix U133 Plus 2.0	54,675	N/A
**B147**	GSM176239	F	"	"	"	"	"	"	"
**B148**	GSM176294	F	"	"	"	"	"	"	"
**B149**	GSM39993	N/A	GALL BLADDER	Institution participating in the study	30 min	GPL1826	SHDP	43,104	[4]
**B150**	GSM2829	N/A	HEART	Commercial human total RNA from donors	N/A	GPL91	Affymetrix U95A	12,626	[2]
**B151**	GSM2856	"	"	"	"	"	"	"	"
**B152**	GSM18792	"	"	"	"	GPL1074	GNF1H	22,639	[3]
**B153**	GSM18793	"	"	"	"	"	"	"	"
**B154**	GSM18951	"	"	"	"	GPL96	Affimetrix U133A	22,283	"
**B155**	GSM18952	"	"	"	"	"	"	"	"
**B156**	GSM39975	"	"	Institution participating in the study	30 min	GPL1825	SHBA	44,160	[4]
**B157**	GSM39980	"	"	"	"	"	"	"	"
**B158**	GSM39992	"	"	"	"	GPL1826	SHDP	43,104	
**B159**	GSM39994	"	"	"	"	"	"	"	"

**B160**	GSM39999	"	"	"	"	"	"	"	"
**B161**	GSM40001	"	"	"	"	"	"	"	"
**B162**	GSM44671	"	"	Commercial human total RNA from donors	N/A	GPL96	Affimetrix U133A	22,283	[5]
**B163**	GSM52565	"	"	N/A	"	"	"	"	[6, 7]
**B164**	GSM175935	M/F mix	"	Commercial human total RNA from donors	N/A	GPL570	Affimetrix U133 Plus 2.0	54,675	N/A
**B165**	GSM790822	M	"	"	"	GPL4133	Agilent-014850 G4112F	45,220	[9]
**B166**	GSM790831	"	"	"	"	"	"	"	"
**B167**	GSM2830	N/A	KIDNEY	"	N/A	GPL91	Affymetrix U95A	12,626	[2]
**B168**	GSM2843	"	"	"	"	"	"	"	"
**B169**	GSM2871	"	"	"	"	"	"	"	"
**B170**	GSM18796	"	"	"	N/A	GPL1074	GNF1H	22,639	[3]
**B171**	GSM18797	"	"	"	"	"	"	"	"
**B172**	GSM18955	"	"	"	"	GPL96	Affimetrix U133A	22,283	"
**B173**	GSM18956	"	"	"	"	"	"	"	"
**B174**	GSM39915	"	"	Institution participating in the study	30 min	GPL1823	SHBW	43,008	[4]
**B175**	GSM39924	"	"	"	"	GPL1824	SHCN	43,008	"
**B176**	GSM39926	"	"	"	"	"	"	"	"
**B177**	GSM39956	"	"	"	"	"	"	"	"
**B178**	GSM39983	"	"	"	"	GPL1825	SHBA	44,160	"
**B179**	GSM44675	"	"	Commercial human total RNA from donors	N/A	GPL96	Affimetrix U133A	22,283	[5]
**B180**	GSM52566	"	"	N/A		"	"	"	[6, 7]
**B181**	GSM175911	M/F mix	"	Commercial human total RNA from donors	N/A	GPL570	Affimetrix U133 Plus 2.0	54,675	N/A
**B182**	GSM774049	N/A	"	N/A	"	GPL96	Affimetrix U133A	22,283	[10]
**B183**	GSM774050	"	"	"	"	"	"	"	"
**B184**	GSM2831	"	LIVER	Commercial human total RNA from donors	"	GPL91	Affymetrix U95A	12,626	[2]
**B185**	GSM2844	"	"	"	"	"	"	"	"
**B186**	GSM18794	"	"	"	"	GPL1074	GNF1H	22,639	[3]
**B187**	GSM18795	"	"	"	"	"	"	"	"
**B188**	GSM18953	"	"	"	"	GPL96	Affimetrix U133A	22,283	"
**B189**	GSM18954	"	"	"	"	"	"	"	"
**B190**	GSM39947	"	"	Institution participating in the study	30 min	GPL1824	SHCN	43,008	[4]
**B191**	GSM39948	"	"	"	"	"	"	"	"

**B192**	GSM39968	"	"	"	"	GPL1825	SHBA	44,160	"
**B193**	GSM39976	"	"	"	"	"	"	"	"
**B194**	GSM39984	"	"	"	"	"	"	"	"
**B195**	GSM44702	"	"	Commercial human total RNA from donors	N/A	GPL96	Affimetrix U133A	22,283	[5]
**B196**	GSM52567	"	"	N/A	"	"	"	"	[6, 7]
**B197**	GSM80728	M	"	Institution participating in the study	8.5 h	GPL570	Affimetrix U133 Plus 2.0	54,675	[1]
**B198**	GSM80729	"	"	"	"	"	"	"	"
**B199**	GSM80730	F	"	"	"	"	"	"	"
**B200**	GSM80739	M	"	"	"	"	"	"	"
**B201**	GSM88581	N/A	"	N/A	N/A	GPL1426	ABI Version 1	33,202	N/A
**B202**	GSM88582	"	"	"	"	"	"	"	"
**B203**	GSM88584	"	"	"	"	"	"	"	"
**B204**	GSM88583	"	"	"	"	"	"	"	"
**B205**	GSM89148	"	"	"	"	GPL1708	Agilent-012391 G4112A	44,290	N/A
**B206**	GSM89149	"	"	"	"	"	"	"	"
**B207**	GSM89150	"	"	"	"	"	"	"	"
**B208**	GSM89151	"	"	"	"	"	"	"	"
**B209**	GSM176332	M	"	Commercial human total RNA from donors	N/A	"	"	"	N/A
**B210**	GSM176333	"	"	"	"	"	"	"	"
**B211**	GSM176334	F	"	"	"	"	"	"	"
**B212**	GSM176335	M	"	"	"	"	"	"	"
**B213**	GSM774051	N/A	"	N/A	"	GPL96	Affimetrix U133A	22,283	[10]
**B214**	GSM774052	"	"	"	"	"	"	"	"
**B215**	GSM790823	M	"	Commercial human total RNA from donors	"	GPL4133	Agilent-014850 G4112F	45,220	[9]
**B216**	GSM790832	"	"	"	"	"	"	"	"
**B217**	GSM2822	N/A	LUNG	"	"	GPL91	Affymetrix U95A	12,626	[2]
**B218**	GSM2832	"	"	"	"	"	"	"	"
**B219**	GSM18790	"	"	"	"	GPL1074	GNF1H	22,639	[3]
**B220**	GSM18791	"	"	"	"	"	"	"	"
**B221**	GSM18949	"	"	"	"	GPL96	Affimetrix U133A	22,283	[9]
**B222**	GSM18950	"	"	"	"	"	"	"	"
**B223**	GSM39909	"	"	Institution participating in the study	30 min	GPL1823	SHBW	43,008	[4]
**B224**	GSM39912	"	"	"	"	"	"	"	"

**B225**	GSM39921	"	"	"	"	"	"	"	"
**B226**	GSM39969	"	"	"	"	GPL1825	SHBA	44,160	"
**B227**	GSM44704	"	"	Commercial human total RNA from donors	N/A	GPL96	Affimetrix U133A	22,283	[5]
**B228**	GSM52568	"	"	N/A	"	"	"	"	[6, 7]
**B229**	GSM80707	"	"	Institution participating in the study	8.5 h	GPL570	Affimetrix U133 Plus 2.0	54,675	[1]
**B230**	GSM80710	"	"	"	"	"	"	"	"
**B231**	GSM80712	"	"	"	"	"	"	"	"
**B232**	GSM88585	"	"	N/A	N/A	GPL1426	ABI Version 1	33,202	N/A
**B233**	GSM88586	"	"	"	"	"	"	"	"
**B234**	GSM88587	"	"	"	"	"	"	"	"
**B235**	GSM88588	"	"	"	"	"	"	"	"
**B236**	GSM89152	"	"	"	"	GPL1708	Agilent-012391 G4112A	44,290	N/A
**B237**	GSM89153	"	"	"	"	"	"	"	"
**B238**	GSM89154	"	"	"	"	"	"	"	"
**B239**	GSM89155	"	"	"	"	"	"	"	"
**B240**	GSM140185	M	"	"	"	GPL201	Affymetrix Human HG-Focus Target Array	8,793	[12]
**B241**	GSM140186	"	"	"	"	"	"	"	"
**B242**	GSM140187	"	"	"	"	"	"	"	"
**B243**	GSM140188	F	"	"	"	"	"	"	"
**B244**	GSM140189	"	"	"	"	"	"	"	"
**B245**	GSM175977	"	"	Commercial human total RNA from donors	N/A	GPL570	Affimetrix U133 Plus 2.0	54,675	N/A
**B246**	GSM176012	M	"	"	"	"	"	"	"
**B247**	GSM176021	"	"	"	"	"	"	"	"
**B248**	GSM774053	N/A	"	N/A	"	GPL96	Affimetrix U133A	22,283	[10]
**B249**	GSM774054	"	"	"	"	"	"	"	"
**B250**	GSM18744	N/A	LYMPHNODE	Commercial human total RNA from donors	N/A	GPL1074	GNF1H	22,639	N/A
**B251**	GSM18745	"	"	"	"	"	"	"	"
**B252**	GSM18903	"	"	"	"	GPL96	Affimetrix U133A	22,283	[3]
**B253**	GSM18904	"	"	"	"	"	"	"	"
**B254**	GSM39930	"	"	Institution participating in the study	30 min	GPL1824	SHCN	43,008	[4]
**B255**	GSM39933	"	"	"	"	"	"	"	"
**B256**	GSM39941	"	"	"	"	"	"	"	"
**B257**	GSM39950	"	"	"	"	"	"	"	"

**B258**	GSM39954	"	"	"	"	"	"	"	"
**B259**	GSM80735	"	"	N/A	N/A	GPL570	Affimetrix U133 Plus 2.0	54,675	[1]
**B260**	GSM80736	"	"	"	"	"	"	"	"
**B261**	GSM80737	"	"	"	"	"	"	"	"
**B262**	GSM80738	"	"	"	"	"	"	"	"
**B263**	GSM176431	M	"	"	"	"	"	"	N/A
**B264**	GSM176432	"	"	"	"	"	"	"	"
**B265**	GSM176433	F	"	"	"	"	"	"	"
**B266**	GSM176434	M	"	"	"	"	"	"	"
**B267**	GSM80716	F	MAMMARY GLAND	Institution participating in the study	8.5 h	"	"	"	[1]
**B268**	GSM80725	"	"	"	"	"	"	"	"
**B269**	GSM80726	"	"	"	"	"	"	"	"
**B270**	GSM176122	"	"	Commercial human total RNA from donors	N/A	"	"	"	N/A
**B271**	GSM176231	"	"	"	"	"	"	"	"
**B272**	GSM176232	"	"	"	"	"	"	"	"
**B273**	GSM80776	M	ORAL MUCOSA	Institution participating in the study	8.5 h	"	"	"	[1]
**B274**	GSM80777	"	"	"	"	"	"	"	"
**B275**	GSM80778	F	"	"	"	"	"	"	"
**B276**	GSM80779	M	"	"	"	"	"	"	"
**B277**	GSM176385	"	"	Commercial human total RNA from donors	N/A	"	"	"	N/A
**B278**	GSM176386	"	"	"	"	"	"	"	"
**B279**	GSM176387	F	"	"	"	"	"	"	"
**B280**	GSM176388	M	"	"	"	"	"	"	"
**B281**	GSM2851	F	OVARY	"	"	GPL91	Affymetrix U95A	12,626	[2]
**B282**	GSM2867	"	"	"	"	"	"	"	"
**B283**	GSM2870	"	"	"	"	"	"	"	"
**B284**	GSM18838	"	"	"	"	GPL1074	GNF1H	22,639	[3]
**B285**	GSM18839	"	"	"	"	"	"	"	"
**B286**	GSM18997	"	"	"	"	GPL96	Affimetrix U133A	22,283	"
**B287**	GSM18998	"	"	"	"	"	"	"	"
**B288**	GSM39892	"	"	Institution participating in the study	30 min	GPL1823	SHBW	43,008	[4]
**B289**	GSM39897	"	"	"	"	"	"	"	"
**B290**	GSM39917	"	"	"	"	"	"	"	"

**B291**	GSM39977	"	"	"	"	GPL1825	SHBA	44,160	
**B292**	GSM39981	"	"	"	"	"	"	"	"
**B293**	GSM44674	"	"	Commercial human total RNA from donors	N/A	GPL96	Affimetrix U133A	22,283	[5]
**B294**	GSM80757	"	"	Institution participating in the study	8.5 h	GPL570	Affimetrix U133 Plus 2.0	54,675	[1]
**B295**	GSM80758	"	"	"	"	"	"	"	"
**B296**	GSM80759	"	"	"	"	"	"	"	"
**B297**	GSM80780	"	"	"	"	"	"	"	"
**B298**	GSM175789	F	"	Commercial human total RNA from donors	N/A	"	"	"	"
**B299**	GSM176131	"	"	"	"	"	"	"	N/A
**B300**	GSM176136	"	"	"	"	"	"	"	N/A
**B301**	GSM176237	"	"	"	"	"	"	"	"
**B302**	GSM176318	"	"	"	"	"	"	"	"
**B303**	GSM790824	"	"	"	8.5 h	GPL4133	Agilent-014850 G4112F	45,220	[9]
**B304**	GSM790833	"	"	"	"	"	"	"	"
**B305**	GSM2845	N/A	PANCREAS	"	N/A	GPL91	Affymetrix U95A	12,626	[2]
**B306**	GSM2872	"	"	"	"	"	"	"	"
**B307**	GSM18818	"	"	"	"	GPL1074	GNF1H	22,639	[3]
**B308**	GSM18819	"	"	"	"	"	"	"	"
**B309**	GSM18977	"	"	"	"	GPL96	Affimetrix U133A	22,283	"
**B310**	GSM18978	"	"	"	"	"	"	"	"
**B311**	GSM39957	"	"	Institution participating in the study	30 min	GPL1824	SHCN	43,008	[4]
**B312**	GSM39990	"	"	"	"	GPL1825	SHBA	44,160	"
**B313**	GSM44677	"	"	Commercial human total RNA from donors	N/A	GPL96	Affimetrix U133A	22,283	[5]
**B314**	GSM52559	"	"	N/A	"	"	"	"	[6, 7]
**B315**	GSM175950	"	"	Commercial human total RNA from donors	"	GPL570	Affimetrix U133 Plus 2.0	54,675	N/A
**B316**	GSM39925	N/A	PARATHYROID	Institution participating in the study	30 min	GPL1824	SHCN	43,008	[4]
**B317**	GSM39940	"	"	"	"	"	"	"	"
**B318**	GSM39953	"	"	"	"	"	"	"	"
**B319**	GSM175881	M	PENIS	Commercial human total RNA from donors	N/A	GPL570	Affimetrix U133 Plus 2.0	54,675	N/A
**B320**	GSM176270	"	"	"	"	"	"	"	"
**B321**	GSM176271	"	"	"	"	"	"	"	"
**B322**	GSM176272	"	"	"	"	"	"	"	"

**B323**	GSM176273	"	"	"	"	"	"	"	"
**B324**	GSM176274	"	"	"	"	"	"	"	"
**B325**	GSM39987	N/A	PERICARDIUM	Institution participating in the study	PMI 30 min	GPL1825	SHBA	44,160	[4]
**B326**	GSM176035	"	"	Commercial human total RNA from donors	N/A	GPL570	Affimetrix U133 Plus 2.0	54,675	N/A
**B327**	GSM80748	M	PHARYNGEAL MUCOSA	Institution participating in the study	8.5 h	"	"	"	[1]
**B328**	GSM80749	F	"	"	"	"	"	"	"
**B329**	GSM80750	M	"	"	"	"	"	"	"
**B330**	GSM80751	"	"	"	"	"	"	"	"
**B331**	GSM175991	"	"	Commercial human total RNA from donors	N/A	"	"	"	N/A
**B332**	GSM175992	F	"	"	"	"	"	"	"
**B333**	GSM176013	M	"	"	"	"	"	"	"
**B334**	GSM176025	"	"	"	"	"	"	"	"
**B335**	GSM2879	N/A	PITUITARY GLAND	"	"	GPL91	Affymetrix U95A	12,626	[2]
**B336**	GSM2898	"	"	"	"	"	"	"	"
**B337**	GSM44699	"	"	"	N/A	GPL96	Affimetrix U133A	22,283	[5]
**B338**	GSM80800	"	"	Institution participating in the study	8.5 h	GPL570	Affimetrix U133 Plus 2.0	54,675	[1]
**B339**	GSM80801	"	"	"	"	"	"	"	"
**B340**	GSM80802	"	"	"	"	"	"	"	"
**B341**	GSM80803	"	"	"	"	"	"	"	"
**B342**	GSM80804	"	"	"	"	"	"	"	"
**B343**	GSM80817	"	"	"	"	"	"	"	"
**B344**	GSM80818	"	"	"	"	"	"	"	"
**B345**	GSM80819	"	"	"	"	"	"	"	"
**B346**	GSM176295	F	"	Commercial human total RNA from donors	N/A	"	"	"	N/A
**B347**	GSM176296	"	"	"	"	"	"	"	"
**B348**	GSM176297	"	"	"	"	"	"	"	"
**B349**	GSM176411	M	"	"	"	"	"	"	"
**B350**	GSM176412	F	"	"	"	"	"	"	"
**B351**	GSM176413	M	"	"	"	"	"	"	"
**B352**	GSM2864	M	PROSTATE	"	N/A	GPL91	Affymetrix U95A	12,626	[2]
**B353**	GSM2865	"	"	"	"	"	"	"	"
**B354**	GSM2866	"	"	"	"	"	"	"	"
**B355**	GSM18798	"	"	"	"	GPL1074	GNF1H	22,639	[3]

**B356**	GSM18799	"	"	"	"	"	"	"	"
**B357**	GSM18957	"	"	"	"	GPL96	Affimetrix U133A	22,283	
**B358**	GSM18958	"	"	"	"	"	"	"	"
**B359**	GSM39910	"	"	Institution participating in the study	30 min	GPL1823	SHBW	43,008	[4]
**B360**	GSM39911	"	"	"	"	"	"	"	"
**B361**	GSM39913	"	"	"	"	"	"	"	"
**B362**	GSM39916	"	"	"	"	"	"	"	"
**B363**	GSM39982	"	"	"	"	GPL1825	SHBA	44,160	"
**B364**	GSM44678	"	"	Commercial human total RNA from donors	N/A	GPL96	Affimetrix U133A	22,283	[5]
**B365**	GSM52560	"	"	N/A	"	"	"	"	[6, 7]
**B366**	GSM80805	"	"	Institution participating in the study	8.5 h	GPL570	Affimetrix U133 Plus 2.0	54,675	[1]
**B367**	GSM80806	"	"	"	"	"	"	"	"
**B368**	GSM80824	"	"	"	"	"	"	"	"
**B369**	GSM175923	"	"	Commercial human total RNA from donors	N/A	"	"	"	N/A
**B370**	GSM175924	"	"	"	"	"	"	"	"
**B371**	GSM175925	"	"	"	"	"	"	"	"
**B372**	GSM175926	"	"	"	"	"	"	"	"
**B373**	GSM175927	"	"	"	"	"	"	"	"
**B374**	GSM175928	"	"	"	"	"	"	"	"
**B375**	GSM175938	"	"	"	"	"	"	"	"
**B376**	GSM175955	"	"	"	"	"	"	"	"
**B377**	GSM176277	"	"	"	"	"	"	"	"
**B378**	GSM176278	"	"	"	"	"	"	"	"
**B379**	GSM176325	"	"	"	"	"	"	"	"
**B380**	GSM176326	"	"	"	"	"	"	"	"
**B381**	GSM176327	"	"	"	"	"	"	"	"
**B382**	GSM774058	"	"	"	"	GPL96	Affimetrix U133A	22,283	[10]
**B383**	GSM2834	M	SALIVARY GLAND	"	"	GPL91	Affymetrix U95A	12,626	[2]
**B384**	GSM2847	"	"	"	"	"	"	"	"
**B385**	GSM18832	"	"	Commercial human total RNA from donors	N/A	GPL1074	GNF1H	22,639	[3]
**B386**	GSM18833	"	"	"	"	"	"	"	"
**B387**	GSM18991	"	"	"	"	GPL96	Affimetrix U133A	22,283	"
**B388**	GSM18992	"	"	"	"	"	"	"	"

**B389**	GSM39891	"	"	Institution participating in the study	30 min	GPL1823	SHBW	43,008	[4]
**B390**	GSM39900	"	"	"	"	"	"	"	"
**B391**	GSM39901	"	"	"	"	"	"	"	"
**B392**	GSM39920	"	"	"	"	"	"	"	"
**B393**	GSM44687	"	"	Commercial human total RNA from donors	N/A	GPL96	Affimetrix U133A	22,283	[5]
**B394**	GSM80820	"	"	Institution participating in the study	8.5 h	GPL570	Affimetrix U133 Plus 2.0	54,675	[1]
**B395**	GSM80821	"	"	"	"	"	"	"	"
**B396**	GSM80822	"	"	"	"	"	"	"	"
**B397**	GSM80823	"	"	"	"	"	"	"	"
**B398**	GSM175939	M/F mix	"	Commercial human total RNA from donors	N/A	"	"	"	N/A
**B399**	GSM176414	M	"	"	"	"	"	"	"
**B400**	GSM176415	"	"	"	"	"	"	"	"
**B401**	GSM176416	F	"	"	"	"	"	"	"
**B402**	GSM176417	M	"	"	"	"	"	"	"
**B403**	GSM44676	N/A	SKELETAL MUSCLE	"	N/A	GPL96	Affimetrix U133A	22,283	[5]
**B404**	GSM52569	"	"	N/A		"	"	"	[6, 7]
**B405**	GSM80790	M	"	Institution participating in the study	8.5 h	GPL570	Affimetrix U133 Plus 2.0	54,675	[1]
**B406**	GSM80791	F	"	"	"	"	"	"	"
**B407**	GSM80792	M	"	"	"	"	"	"	"
**B408**	GSM80796	"	"	"	"	"	"	"	"
**B409**	GSM80797	F	"	"	"	"	"	"	"
**B410**	GSM175882	M	"	Commercial human total RNA from donors	N/A	"	"	"	N/A
**B411**	GSM175883	F	"	"	"	"	"	"	"
**B412**	GSM175884	M	"	"	"	"	"	"	"
**B413**	GSM175940	M/F mix	"	"	"	"	"	"	"
**B414**	GSM175985	M	"	"	"	"	"	"	"
**B415**	GSM774056	N/A	"	"	"	GPL96	Affimetrix U133A	22,283	[10]
**B416**	GSM774057	"	"	"	"	"	"	"	"
**B417**	GSM790826	M	"	"	"	GPL4133	Agilent-014850 G4112F	45,220	[9]
**B418**	GSM790835	"	"	"	"	"	"	"	"
**B419**	GSM18842	N/A	SKIN	"	"	GPL1074	GNF1H	22,639	[3]
**B420**	GSM18843	"	"	"	"	"	"	"	"
**B421**	GSM19001	"	"	"	"	GPL96	Affimetrix U133A	22,283	"

**B422**	GSM19002	"	"	"	"	"	"	"	"
**B423**	GSM44686	"	"	"	"	"	"	"	[5]
**B424**	GSM52561	"	"	N/A		"	"	"	[6, 7]
**B425**	GSM175948	M/F mix	"	Commercial human total RNA from donors	N/A	GPL570	Affimetrix U133 Plus 2.0	54,675	N/A
**B426**	GSM175952	N/A	"	"	"	"	"	"	"
**B427**	GSM175967	M	"	"	"	"	"	"	"
**B428**	GSM175993	"	"	"	"	"	"	"	"
**B429**	GSM175998	N/A	"	"	"	"	"	"	"
**B430**	GSM176117	M	"	"	"	"	"	"	"
**B431**	GSM176267	F	"	"	"	"	"	"	"
**B432**	GSM44679	N/A	SMALL INTESTINE	"	"	GPL96	Affimetrix U133A	22,283	[5]
**B433**	GSM52562	"	"	N/A	"	"	"	"	[6, 7]
**B434**	GSM175908	M/F mix	"	"	"	GPL570	Affimetrix U133 Plus 2.0	54,675	N/A
**B435**	GSM175947	"	"	"	"	"	"	"	"
**B436**	GSM774059	N/A	"	"	"	GPL96	Affimetrix U133A	22,283	[10]
**B437**	GSM774060	"	"	"	"	"	"	"	"
**B438**	GSM18812	N/A	SMOOTH MUSCLE	Commercial human total RNA from donors	"	GPL1074	GNF1H	22,639	[3]
**B439**	GSM18813	"	"	"	"	"	"	"	"
**B440**	GSM18971	"	"	"	"	GPL96	Affimetrix U133A	22,283	"
**B441**	GSM18972	"	"	"	"	"	"	"	"
**B442**	GSM2852	N/A	SPINAL CORD	"	"	GPL91	Affymetrix U95A	12,626	[2]
**B443**	GSM2855	"	"	"	"	"	"	"	"
**B444**	GSM18784	"	"	"	"	GPL1074	GNF1H	22,639	[3]
**B445**	GSM18785	"	"	"	"	"	"	"	"
**B446**	GSM18943	"	"	"	"	GPL96	Affimetrix U133A	22,283	"
**B447**	GSM18944	"	"	"	"	"	"	"	"
**B448**	GSM44700	"	"	"	"	"	"	"	[5]
**B449**	GSM80784	"	"	Institution participating in the study	8.5 h	GPL570	Affimetrix U133 Plus 2.0	54,675	[1]
**B450**	GSM80785	"	"	"	"	"	"	"	"
**B451**	GSM80786	"	"	"	"	"	"	"	"
**B452**	GSM80787	"	"	"	"	"	"	"	"
**B453**	GSM80794	"	"	"	"	"	"	"	"
**B454**	GSM80795	"	"	"	"	"	"	"	"
**B455**	GSM80798	"	"	"	"	"	"	"	"

**B456**	GSM80799	"	"	"	"	"	"	"	"
**B457**	GSM175865	M	"	Commercial human total RNA from donors	N/A	"	"	"	N/A
**B458**	GSM175866	"	"	"	"	"	"	"	"
**B459**	GSM175867	F	"	"	"	"	"	"	"
**B460**	GSM175868	M	"	"	"	"	"	"	"
**B461**	GSM175936	M/F mix	"	"	"	"	"	"	"
**B462**	GSM176057	M	"	"	"	"	"	"	"
**B463**	GSM176074	F	"	"	"	"	"	"	"
**B464**	GSM176208	"	"	"	"	"	"	"	"
**B465**	GSM176209	"	"	"	"	"	"	"	"
**B466**	GSM2835	N/A	SPLEEN	"	"	GPL91	Affymetrix U95A	12,626	[2]
**B467**	GSM2858	"	"	"	"	"	"	"	"
**B468**	GSM39934	"	"	Institution participating in the study	30 min	GPL1824	SHCN	43,008	[4]
**B469**	GSM39963	"	"	"	"	GPL1825	SHBA	44,160	
**B470**	GSM39971	"	"	"	"	"	"	"	"
**B471**	GSM44673	"	"	Commercial human total RNA from donors	N/A	GPL96	Affimetrix U133A	22,283	[5]
**B472**	GSM52570	"	"	N/A		"	"	"	[6, 7]
**B473**	GSM80807	"	"	Institution participating in the study	8.5 h	GPL570	Affimetrix U133 Plus 2.0	54,675	[1]
**B474**	GSM80808	"	"	"	"	"	"	"	"
**B475**	GSM80825	"	"	"	"	"	"	"	"
**B476**	GSM80826	"	"	"	"	"	"	"	"
**B477**	GSM175941	M/F mix	"	Commercial human total RNA from donors	N/A	"	"	"	N/A
**B478**	GSM176328	M	"	"	"	"	"	"	"
**B479**	GSM176329	"	"	"	"	"	"	"	"
**B480**	GSM176330	F	"	"	"	"	"	"	"
**B481**	GSM176331	M	"	"	"	"	"	"	"
**B482**	GSM774061	N/A	"	N/A	"	GPL96	Affimetrix U133A	22,283	[10]
**B483**	GSM774062	"	"	"		"	"	"	
**B484**	GSM44703	"	STOMACH	Commercial human total RNA from donors	"	"	"	"	[5]
**B485**	GSM52557	"	"	N/A	"	"	"	"	[6, 7]
**B486**	GSM175943	M/F mix	"	Commercial human total RNA from donors	"	GPL570	Affimetrix U133 Plus 2.0	54,675	N/A
**B487**	GSM774063	N/A	"	"	"	GPL96	Affimetrix U133A	22,283	[10]

**B488**	GSM774064	"	"	"	"	"	"	"	"
**B489**	GSM175810	N/A	SYNOVIAL MEMBRANE	"	N/A	GPL570	Affimetrix U133 Plus 2.0	54,675	N/A
**B490**	GSM175811	"	"	"	"	"	"	"	"
**B491**	GSM175812	"	"	"	"	"	"	"	"
**B492**	GSM176290	"	"	"	"	"	"	"	"
**B493**	GSM176291	"	"	"	"	"	"	"	"
**B494**	GSM176292	"	"	"	"	"	"	"	"
**B495**	GSM2823	M	TESTIS	"	"	GPL91	Affymetrix U95A	12,626	[2]
**B496**	GSM2824	"	"	"	"	"	"	"	"
**B497**	GSM18822	"	"	"	"	GPL1074	GNF1H	22,639	[3]
**B498**	GSM18823	"	"	"	"	"	"	"	"
**B499**	GSM18981	"	"	"	"	GPL96	Affimetrix U133A	22,283	"
**B500**	GSM18982	"	"	"	"	"	"	"	"
**B501**	GSM39903	"	"	Institution participating in the study	30 min	GPL1823	SHBW	43,008	[4]
**B502**	GSM39958	"	"	"	"	GPL1824	SHCN	43,008	"
**B503**	GSM39973	"	"	"	"	GPL1825	SHBA	44,160	"
**B504**	GSM44701	"	"	Commercial human total RNA from donors	N/A	GPL96	Affimetrix U133A	22,283	[5]
**B505**	GSM80853	"	"	Institution participating in the study	8.5 h	GPL570	Affimetrix U133 Plus 2.0	54,675	[1]
**B506**	GSM80868	"	"	"	"	"	"	"	"
**B507**	GSM80869	"	"	"	"	"	"	"	"
**B508**	GSM175942	"	"	Commercial human total RNA from donors	N/A	"	"	"	N/A
**B509**	GSM176113	"	"	"	"	"	"	"	"
**B510**	GSM176275	"	"	"	"	"	"	"	"
**B511**	GSM176276	"	"	"	"	"	"	"	"
**B512**	GSM176422	"	"	"	"	"	"	"	"
**B513**	GSM176423	"	"	"	"	"	"	"	"
**B514**	GSM774065	"	"	"	"	GPL96	Affimetrix U133A	22,283	[10]
**B515**	GSM774066	"	"	"	"	"	"	"	"
**B516**	GSM790827	"	"	"	"	GPL4133	Agilent-014850 G4112F	45,220	[9]
**B517**	GSM790836	"	"	"	"	"	"	"	"
**B518**	GSM2825	N/A	THYMUS	"	N/A	GPL91	Affymetrix U95A	12,626	[2]
**B519**	GSM2826	"	"	"	"	"	"	"	"
**B520**	GSM18740	"	"	"	"	GPL1074	GNF1H	22,639	[3]
**B521**	GSM18741	"	"	"	"	"	"	"	"

**B522**	GSM18899	"	"	"	"	GPL96	Affimetrix U133A	22,283	"
**B523**	GSM18900	"	"	"	"	"	"	"	"
**B524**	GSM39898	"	"	Institution participating in the study	30 min	GPL1823	SHBW	43,008	[4]
**B525**	GSM39942	"	"	"	"	GPL1824	SHCN	43,008	"
**B526**	GSM44672	"	"	Commercial human total RNA from donors	N/A	GPL96	Affimetrix U133A	22,283	[5]
**B527**	GSM175973	M	"	"	"	GPL570	Affimetrix U133 Plus 2.0	54,675	N/A
**B528**	GSM176262	F	"	"	"	"	"	"	"
**B529**	GSM774067	N/A	"	"	"	GPL96	Affimetrix U133A	22,283	[10]
**B530**	GSM774068	"	"	"	"	"	"	"	"
**B531**	GSM790828	M	"	"	"	GPL4133	Agilent-014850 G4112F	45,220	[9]
**B532**	GSM790837	"	"	"	"	"	"	"	"
**B533**	GSM18858	N/A	TONGUE	"	"	GPL1074	GNF1H	22,639	[3]
**B534**	GSM18859	"	"	"	"	"	"	"	"
**B535**	GSM19017	"	"	"	"	GPL96	Affimetrix U133A	22,283	"
**B536**	GSM19018	"	"	"	"	"	"	"	"
**B537**	GSM80842	"	"	Institution participating in the study	8.5 h	GPL570	Affimetrix U133 Plus 2.0	54,675	[1]
**B538**	GSM80843	"	"	"	"	"	"	"	"
**B539**	GSM80844	"	"	"	"	"	"	"	"
**B540**	GSM80845	"	"	"	"	"	"	"	"
**B541**	GSM175946	M/F mix	"	Commercial human total RNA from donors	N/A	"	"	"	N/A
**B542**	GSM175953	"	"	"	"	"	"	"	"
**B543**	GSM18742	N/A	TONSIL	"	N/A	GPL1074	GNF1H	22,639	[3]
**B544**	GSM18743	"	"	"	"	"	"	"	"
**B545**	GSM18901	"	"	"	"	GPL96	Affimetrix U133A	22,283	"
**B546**	GSM18902	"	"	"	"	"	"	"	"
**B547**	GSM39929	"	"	Institution participating in the study	30 min	GPL1824	SHCN	43,008	[4]
**B548**	GSM39931	"	"	"	"	"	"	"	"
**B549**	GSM39944	"	"	"	"	"	"	"	"
**B550**	GSM39951	"	"	"	"	"	"	"	"
**B551**	GSM80886	"	"	Institution participating in the study	8.5 h	GPL570	Affimetrix U133 Plus 2.0	54,675	[1]
**B552**	GSM80889	"	"	"	"	"	"	"	"
**B553**	GSM80901	"	"	"	"	"	"	"	"
**B554**	GSM175976	F	"	Commercial human	N/A	"	"	"	N/A

				total RNA from donors					
**B555**	GSM176015	M	"	"	"	"	"	"	"
**B556**	GSM176114	"	"	"	"	"	"	"	"
**B557**	GSM2854	N/A	TRACHEA	"	"	GPL91	Affymetrix U95A	12,626	[2]
**B558**	GSM2873	"	"	"	"	"	"	"	"
**B559**	GSM18834	"	"	"	"	GPL1074	GNF1H	22,639	[3]
**B560**	GSM18835	"	"	"	"	"	"	"	"
**B561**	GSM18993	"	"	"	"	GPL96	Affimetrix U133A	22,283	"
**B562**	GSM18994	"	"	"	"	"	"	"	"
**B563**	GSM44688	"	"	"	"	"	"	"	[5]
**B564**	GSM80887	"	"	Institution participating in the study	8.5 h	GPL570	Affimetrix U133 Plus 2.0	54,675	[1]
**B565**	GSM80888	"	"	"	"	"	"	"	"
**B566**	GSM80890	"	"	"	"	"	"	"	"
**B567**	GSM175944	M/F mix	"	Commercial human total RNA from donors	N/A	"	"	"	N/A
**B568**	GSM175980	M	"	"	"	"	"	"	"
**B569**	GSM175981	F	"	"	"	"	"	"	"
**B570**	GSM176023	M	"	"	"	"	"	"	"
**B571**	GSM18846	N/A	TRIGEMINAL GANGLION	"	"	GPL1074	GNF1H	22,639	[3]
**B572**	GSM18847	"	"	"	"	"	"	"	"
**B573**	GSM19005	"	"	"	"	GPL96	Affimetrix U133A	22,283	"
**B574**	GSM19006	"	"	"	"	"	"	"	"
**B575**	GSM80875	"	"	Institution participating in the study	8.5 h	GPL570	Affimetrix U133 Plus 2.0	54,675	[1]
**B576**	GSM80876	"	"	"	"	"	"	"	"
**B577**	GSM80877	"	"	"	"	"	"	"	"
**B578**	GSM80878	"	"	"	"	"	"	"	"
**B579**	GSM80891	"	"	"	"	"	"	"	"
**B580**	GSM80895	"	"	"	"	"	"	"	"
**B581**	GSM80905	"	"	"	"	"	"	"	"
**B582**	GSM80906	"	"	"	"	"	"	"	"
**B583**	GSM175889	M	"	Commercial human total RNA from donors	N/A	"	"	"	N/A
**B584**	GSM175890	"	"	"	"	"	"	"	"
**B585**	GSM175891	F	"	"	"	"	"	"	"
**B586**	GSM175892	M	"	"	"	"	"	"	"
**B587**	GSM176063	"	"	"	"	"	"	"	"

**B588**	GSM176076	F	"	"	"	"	"	"	"
**B589**	GSM176220	"	"	"	"	"	"	"	"
**B590**	GSM176221	"	"	"	"	"	"	"	"
**B591**	GSM80911	N/A	URETHRA	Institution participating in the study	8.5 h	"	"	"	[1]
**B592**	GSM80912	"	"	"	"	"	"	"	"
**B593**	GSM80913	"	"	"	"	"	"	"	"
**B594**	GSM176279	M	"	Commercial human total RNA from donors	N/A	"	"	"	N/A
**B595**	GSM176280	"	"	"	"	"	"	"	"
**B596**	GSM176428	"	"	"	"	"	"	"	"
**B597**	GSM176429	"	"	"	"	"	"	"	"
**B598**	GSM176430	"	"	"	"	"	"	"	"
**B599**	GSM2838	F	UTERUS	"	"	GPL91	Affymetrix U95A	12,626	[2]
**B600**	GSM2853	"	"	"	"	"	"	"	"
**B601**	GSM18800	"	"	"	"	GPL1074	GNF1H	22,639	[3]
**B602**	GSM18801	"	"	"	"	"	"	"	"
**B603**	GSM18959	"	"	"	"	GPL96	Affimetrix U133A	22,283	"
**B604**	GSM18960	"	"	"	"	"	"	"	"
**B605**	GSM44684	"	"	"	"	"	"	"	[5]
**B606**	GSM175945	"	"	"	"	GPL570	Affimetrix U133 Plus 2.0	54,675	N/A
**B607**	GSM39894	F	VAGINA	Institution participating in the study	30 min	GPL1823	SHBW	43,008	"
**B608**	GSM80874	"	"	Institution participating in the study	8.5 h	GPL570	Affimetrix U133 Plus 2.0	54,675	[1]
**B609**	GSM80902	"	"	"	"	"	"	"	"
**B610**	GSM80903	"	"	"	"	"	"	"	"
**B611**	GSM80904	"	"	"	"	"	"	"	"
**B612**	GSM175878	"	"	Commercial human total RNA from donors	N/A	"	"	"	N/A
**B613**	GSM176129	"	"	"	"	"	"	"	"
**B614**	GSM176134	"	"	"	"	"	"	"	"
**B615**	GSM176139	"	"	"	"	"	"	"	"
**B616**	GSM176038	F	VENA CAVA	"	"	"	"	"	"
**B617**	GSM80897	F	VULVA	Institution participating in the study	8.5 h	"	"	"	[1]
**B618**	GSM80898	"	"	"	"	"	"	"	"
**B619**	GSM80899	"	"	"	"	"	"	"	"
**B620**	GSM80900	"	"	"	"	"	"	"	"

**B621**	GSM176078	"	"	Commercial human total RNA from donors	N/A	"	"	"	N/A
**B622**	GSM176079	"	"	"	"	"	"	"	"
**B623**	GSM176080	"	"	"	"	"	"	"	"
**B624**	GSM176081	"	"	"	"	"	"	"	"

From the left to right: our study identifiers (ID); GEO (Gene Expression Omnibus) Sample ID; sex, when it is available; sample type; sample collection; post-mortem interval (PMI) before freezing; GEO Platform name; platform type; platform spot number and GEO experiment citation. All citations indicated as N/A: not available.

REFERENCES

1. Roth RB, Hevezi P, Lee J, Willhite D, Lechner SM, Foster AC, Zlotnik A (2006) Gene expression analyses reveal molecular relationships among 20 regions of the human CNS. Neurogenetics 7(2):67-80. doi: 10.1007/s10048-006-0032-6

2. Su AI, Cooke MP, Ching KA, Hakak Y, Walker JR, Wiltshire T, Orth AP, Vega RG, Sapinoso LM, Moqrich A, Patapoutian A, Hampton GM, Schultz PG, Hogenesch JB (2002) Large-scale analysis of the human and mouse transcriptomes. Proc Natl Acad Sci U S A 99(7):4465-4470. doi: 10.1073/pnas.012025199

3. Su AI, Wiltshire T, Batalov S, Lapp H, Ching KA, Block D, Zhang J, Soden R, Hayakawa M, Kreiman G, Cooke MP, Walker JR, Hogenesch JB (2004) A gene atlas of the mouse and human protein-encoding transcriptomes. Proc Natl Acad Sci U S A 101(16):6062-6067. doi: 10.1073/pnas.0400782101

4. Shyamsundar R, Kim YH, Higgins JP, Montgomery K, Jorden M, Sethuraman A, van de Rijn M, Botstein D, Brown PO, Pollack JR (2005) A DNA microarray survey of gene expression in normal human tissues. Genome Biol 6(3):R22. doi:10.1186/gb-2005-6-3-r22

5. Ge X, Yamamoto S, Tsutsumi S, Midorikawa Y, Ihara S, Wang SM, Aburatani H (2005) Interpreting expression profiles of cancers by genome-wide survey of breadth of expression in normal tissues. Genomics 86(2):127-141. doi: 10.1016/j.ygeno.2005.04.008

6. Detwiller KY, Fernando NT, Segal NH, Ryeom SW, D'Amore PA, Yoon SS (2005) Analysis of hypoxia-related gene expression in sarcomas and effect of hypoxia on RNA interference of vascular endothelial cell growth factor A. Cancer Res 65(13):5881-5889. doi: 10.1158/0008-5472.CAN-04-4078

7. Yoon SS, Segal NH, Park PJ, Detwiller KY, Fernando NT, Ryeom SW, Brennan MF, Singer S (2006) Angiogenic profile of soft tissue sarcomas based on analysis of circulating factors and microarray gene expression. J Surg Res 135(2):282-290. doi: 10.1016/j.jss.2006.01.023

8. Irizarry RA, Wang C, Zhou Y, Speed TP (2009) Gene set enrichment analysis made simple. Stat Methods Med Res 18:565-575. doi: 10.1177/0962280209351908. Erratum in: Stat Methods Med Res 2011 20:571

9. Steinfeld I, Navon R, Ach R, Yakhini Z (2013) miRNA target enrichment analysis reveals directly active miRNAs in health and disease. Nucleic Acids Res 41(3):e45. doi: 10.1093/nar/gks1142

10. Martens GA, Jiang L, Hellemans KH, Stangé G, Heimberg H, Nielsen FC, Sand O, Van Helden J, Van Lommel L, Schuit F, Gorus FK, Pipeleers DG (2011) Clusters of conserved beta cell marker genes for assessment of beta cell phenotype. PLoS One 6(9):e24134. doi: 10.1371/journal.pone.0024134

11. McLachlan JL, Smith AJ, Bujalska IJ, Cooper PR (2005) Gene expression profiling of pulpal tissue reveals the molecular complexity of dental caries. Biochim Biophys Acta 1741(3):271-281. doi: 10.1016/j.bbadis.2005.03.007

12. Rohrbeck A, Neukirchen J, Rosskopf M, Pardillos GG, Geddert H, Schwalen A, Gabbert HE, von Haeseler A, Pitschke G, Schott M, Kronenwett R, Haas R, Rohr UP (2008) Gene expression profiling for molecular distinction and characterization of laser captured primary lung cancers. J Transl Med 6:69. doi: 10.1186/1479-5876-6-69
