# Supplementary material for: A molecular view of the normal human thyroid structure and function reconstructed from its reference transcriptome map
Source: BMC Genomics. 2017 Sep 18;18:739. doi: 10.1186/s12864-017-4049-z (PMC5604164; doi:10.1186/s12864-017-4049-z)
Supplement: Supplementary file 7 — PCR primer pairs used to validate thyroid TRAM map. (PDF 96 kb) [file 12864_2017_4049_MOESM7_ESM.pdf]

Additional file 7: Table S7. Primer pairs used to validate thyroid TRAM map.

**Gene Symbol**	**Gene Name**	**RefSeq RNA GenBank Accession No.**	**Primer pair sequences (5′→ 3′)[a]**	**RT-PCR product size** (base pair)
TG	Thyroglobulin	NM_003235	gtctgaccagggagcgtttag<br>gtccagatgaagctggaatgag	**173**
RPL41	Ribosomal protein L41	NM_001035267	tggaggaagaagcgaatgcgc<br>tcccacaacttgtaccagcatc	**150**
B2M	Beta-2-microglobulin	NM_004048	ctactctctctttctggcctgg<br>cccagacacatagcaattcagg	**114**
ACTB[b]	Actin, beta	NM_001101	gagcatcccccaaagttcacaat<br>cttcctgtaacaacgcatctcata	**99**
TPO	Thyroid peroxidase	NM_000547	tagcgtcttggaggaaagcaag<br>tctgctgctcgggcaatcact	**156**
IYD	Iodotyrosine deiodinase	NM_001164694	tgggtcacagacctcaagaaac<br>atgccacaagcgatggaaacac	**164**
SDC2	Syndecan 2	NM_002998	aggagtgtatcctattgatgacg<br>gtttcttcaggtgactttgtctg	**198**

TSHR	Thyroid stimulating hormone receptor	NM_000369	acttacatagaccctgatgccc<br>gacgtcatgtaagggttgtctg	**155**
SERPINF1[c]	Serpin family F member 1	NM_002615	ggctgtctccaacttcggcta<br>cggtgaatgatggattctgttcg	**150**
MYH9	Myosin, heavy chain 9, non-muscle	NM_002473	gcctacaggagtatgatgcaag<br>tcttgctcttgtgcgaggacg	**136**
BACE2	Beta-site APP-cleaving enzyme 2	NM_012105	ggtcttacttccctaaaatctcc<br>gaaatgccgaatcggtaacattc	**142**
REEP3	Receptor accessory protein 3	NM_001001330	actgtgattgaaacagtagccg<br>tcctttggtatagggagaaagc	**105**
YKT6	YKT6 v-SNARE homolog (*S. cerevisiae*)	NM_006555	tcttgcaggtgtggtcattgc<br>gcctgcactttagtcatggga	**210**
NTNG1[c]	Netrin G1	NM_001113226	gaaagtgaaactcgatcctccg<br>ctcgcatcacactcattattgc	**102**
RIPPLY3	Ripply transcriptional repressor 3	NM_018962	acctagggctgaccaacacac<br>ctccgcaggtattcttgacgc	**105**

NPTX1 [c]	Neuronal pentraxin 1	NM_002522	gggcaaactttgcaatcgctc<br>tctcctcggtgtcgttcctg	**193**
TBX18 [c]	T-box 18	NM_001080508	ctcgggggagacttggatgag<br>ctgattctgataggcagtgacg	**247**

[a]Top: forward primer; bottom: reverse primer (for each gene).

[b]Only for this gene the primers were designed on the same exon using the sequence aligned to the hybridization probe.

[c]These primer pairs were used for the first time in a previous work (Caracausi et al., 2014).
